# Digital EduHealth for the wellbeing of minority university students: a scoping review

**DOI:** 10.3389/fpubh.2026.1781600

**Published:** 2026-03-20

**Authors:** Jessidenes Teixeira de Freitas Mendes Leal, Sara Dias-Trindade, Joilda Silva Nery, Júlio César Leal Pereira, Márcio Santos da Natividade, Rita Carvalho-Sauer, Renata Meira Veras, Ramon da Costa Saavedra, Clarice Santos Mota

**Affiliations:** 1 Instituto de Saúde Coletiva, Universidade Federal da Bahia, Salvador, BA, Brazil; 2 Prefeitura Municipal de Salvador, Secretaria de Saúde, Salvador, BA, Brazil; 3 Centro de Investigação Transdisciplinar “Cultura, Espaço e Memória” (CITCEM), Faculdade de Letras, Universidade do Porto, Porto, Portugal; 4 Florida University of Science and Theology, Pompano Beach, FL, United States; 5 Florida Christian University, Orlando, FL, United States; 6 Governo do Estado da Bahia, Secretaria de Saúde do Estado (SESAB), Salvador, BA, Brazil; 7 Instituto de Humanidades, Artes e Ciências, Universidade Federal da Bahia, Salvador, BA, Brazil

**Keywords:** Digital EduHealth, digital health, health equity, higher education, mental health, minority students, scoping review

## Abstract

**Introduction:**

Inequalities in mental health persist in higher education, disproportionately affecting students from social minorities, despite the progress made by affirmative action policies.

**Methods:**

This scoping review follows the Arksey and O'Malley framework, as refined by Levac and colleagues, and the recommendations of the JBI Manual for Evidence Synthesis for scoping reviews. It includes 55 articles from journals on digital interventions that integrate health and education, focusing on the well-being of university students belonging to minority social groups. This scoping review protocol has been registered with Open Science Framework and can be accessed at https://osf.io/yu76c/?view_only=91260110a2d5410f92731ff29a0eff71.

**Results:**

The results revealed the positive effects of digital interventions, especially those with culturally sensitive approaches. However, barriers such as digital exclusion, institutional racism, and poor infrastructure continue to compromise adherence, effectiveness, and inclusion.

**Discussion:**

While Digital EduHealth promotes emotional well-being and academic belonging, its success relies on cultural sensitivity, continuous support, and intersectional approaches. Overcoming barriers requires intersectoral strategies. Ultimately, integrating digital health and education provides an emancipatory tool to advance equity and university retention, complementing human contact.

**Systematic review registration:**

https://osf.io/yu76c?view_only=91260110a2d5410f92731ff29a0eff71.

## Introduction

Structural inequalities persist as critical determinants of psychosocial wellbeing in higher education, disproportionately affecting students belonging to social minorities, including black, indigenous, immigrant, and LGBTQIA+ individuals. Affirmative action policies have made significant progress in addressing inequalities in access to higher education. However, they alone cannot ensure dignified permanence or protect against the institutional and structural forms of exclusion that pervade the university experience (1, 2). The absence of institutional strategies that promote the wellbeing of these students represents a historical gap in inclusion and care.

Despite advances in inclusion policies, the mental health of university students remains a significant global challenge. Anxiety and/or depression symptoms are prevalent among university students worldwide, reaching alarming proportions in different regions: 56% in North America, 54% in South America, 51% in Europe, 40% in Africa, 33% in Asia, and 19% in Oceania. This results in a global average prevalence of 37% (3–5). In light of this, there is an urgent need to implement institutional strategies that promote wellbeing at universities, particularly for vulnerable groups, such as black students who benefit from affirmative action (3–6).

Recognized by the World Health Organization as the application of digital tools to expand access to care and wellbeing, digital health is an interdisciplinary field that brings together medical informatics, public health, health management, and technological innovation. Its applications range from large-scale data mapping to individual care mediated by mobile devices, call center platforms, and interoperable information systems (7). When linked to educational strategies, these technologies increase their potential to promote equity and inclusion.

The European Union Action Plan defines digital education as the integration of digital technologies to improve access to and the quality of learning. It also involves developing digital skills in learners so they can live, work, and thrive in technologized societies (8). With this in mind, the educational environment can and should be a space for digital care as long as it is supported by robust public policies.

This article introduces the concept of Digital EduHealth, defined as the integration of educational components into health-related digital technologies to promote the psychosocial wellbeing of university students. The concept encompasses interventions, the development of digital skills, equitable access to technological tools, and the creation of psychosocial and educational support environments mediated by digital solutions. Although it is centered on digital health, it recognizes the educational environment as a strategic space for care. Examples include emotional self-care platforms, telepsychology, academic support applications with a psychosocial focus, and institutional virtual environments aimed at student retention. The concept is anchored in principles that reconfigure teaching and care for the digital age. It requires collaboration between educators, the private sector, civil society, and authorities to promote quality, inclusion, and accessibility. Its unique feature lies in intentionally articulating digital care strategies with the promotion of equity in higher education, where health and education are integrated determinants of university retention. Additionally, it aligns with the vision of equitably strengthening health systems through digitization and interoperability by providing the sector with the necessary foundations, tools, and platforms for digital transformation (9–12).

Black students and students from other ethnic minorities face multiple barriers to accessing mental health services. These barriers include a low supply of culturally competent services, fear of stigmatization, and perceptions of institutional exclusion (13, 14). In Brazil, quota students experience symbolic racism, academic isolation, and subjective suffering even after being admitted through affirmative action (1, 15).

The COVID-19 pandemic accentuated these inequalities by accelerating the digitization of services without adequate institutional preparation. Reports from students and teachers at public universities point to an increase in symptoms of anxiety, overload, and difficulty engaging in remote learning, particularly among the most vulnerable (16, 17). Digital exclusion, characterized by a lack of access to equipment, connectivity, and digital skills, exacerbates these vulnerabilities (18).

Digital transformation has proven to be an international priority. In South Africa, for instance, national strategies present e-health and e-education policies as instruments of equity when accompanied by investments in infrastructure and training (9, 19). In the European Union, Regulation (EU) 2021/522 outlines guidelines for digital health transformation with a focus on equity, interoperability, and active user participation (20). Brazil is promoting the digital transformation of health in pursuit of consolidated interoperability (21) and emphasizing the inclusion of digital practices at all educational levels to promote equal access to digital education (22, 23).

This scoping review aims to map the available evidence on how Digital EduHealth interventions promote the wellbeing of university students belonging to social minorities. By doing so, the review seeks to contribute to the development of digitally innovative and inequality-sensitive policies in higher education that align with the Sustainable Development Goals of the 2030 Agenda and the WHO Global Strategy on Digital Health (11, 24).

## Methods

### Methodological structure

This scoping review was conducted according to the methodological framework proposed by Arksey and O'Malley (25) and improved upon by Levac et al. (26). It also followed the recommendations of the JBI Handbook for Scoping Reviews (27) and is aligned with the PRISMA-ScR (Preferred Reporting Items for Systematic Reviews and Meta-Analyses Extension for Scoping Reviews) (28) checklist, which is available in Supplementary material. The review protocol was previously registered on the Open Science Framework (OSF) platform, which is a free, open digital infrastructure for the transparent registration and sharing of scientific research projects. The link to the research protocol on OSF is https://osf.io/yu76c/?view_only=91260110a2d5410f92731ff29a0eff71.

### Research question

The research question was structured based on the PCC (Population, Concept, Context) model and covered university students belonging to social minorities (population), Digital EduHealth interventions (concept), and wellbeing in higher education (context). Thus, the guiding question was: Which Digital EduHealth interventions promote the wellbeing of university students belonging to social minorities?

Here, the concept of social minorities refers to groups that have historically been vulnerable to institutional and symbolic exclusion, regardless of their numerical representation in society (29). For this review, black, indigenous, immigrant, low-income, and LGBTQIA+ students are considered social minorities because they are often the target of affirmative action policies.

### Search strategy

The search was conducted between May and June of 2025. It covered studies worldwide in the PubMed/MEDLINE, Embase, Scopus, Web of Science, and PsycINFO databases. The search was supplemented by gray literature identified in Google Scholar. Descriptors and keywords were defined based on a preliminary literature review and adapted to the specificities of each database. The complete search strategy is available in Supplementary material.

### Inclusion and exclusion criteria

Studies published in English, Portuguese, or Spanish between January 2015 and April 2025 were included if they addressed digital interventions integrated into educational and health contexts aimed at university students belonging to social minorities and presented results related to the psychosocial wellbeing of these students worldwide. The 2015 start date is based on the consolidation of public policies and institutional strategies aimed at digitizing health and education during this period. Examples include the World Health Organization's Global Strategy on Digital Health and the advancement of digital education platforms, particularly in higher education. These developments have driven the creation of integrated interventions focused on wellbeing and inclusion (8, 11).

Studies outside the context of higher education were excluded, as were studies that did not address digital interventions integrated into healthcare and education. Other excluded studies include non-original works (e.g., reviews, protocols, editorials, book chapters, comments, letters, and event summaries); studies published in languages other than English, Portuguese, or Spanish; and works without empirical results on psychosocial wellbeing or that focus on other population groups.

### Study selection

After removing duplicates, a lead reviewer screened the titles and abstracts based on previously defined eligibility criteria using the Rayyan platform to manage, organize, and track the screening process (30). Rayyan is a digital tool designed specifically for systematic reviews (though it is also useful for other types of reviews, such as scoping reviews) that allows for reviewer blinding, facilitates conflict resolution, and increases transparency in study selection. Any conflicts regarding potentially relevant studies were resolved by a second reviewer after a full-text evaluation. The selection process will be presented in a flowchart according to the PRISMA-ScR guidelines.

### Data extraction and synthesis

Data from the included studies were extracted using a standardized form in an Excel spreadsheet. This form was initially structured on the Rayyan platform and later supplemented with items related to the characteristics of the interventions, the population, the reported results, the effects, the barriers, the facilitators, the implications, and the institutional context. The synthesis will predominantly be descriptive, with thematic analysis identifying patterns and recurring elements in the interventions.

## Results

**Figure 1:**
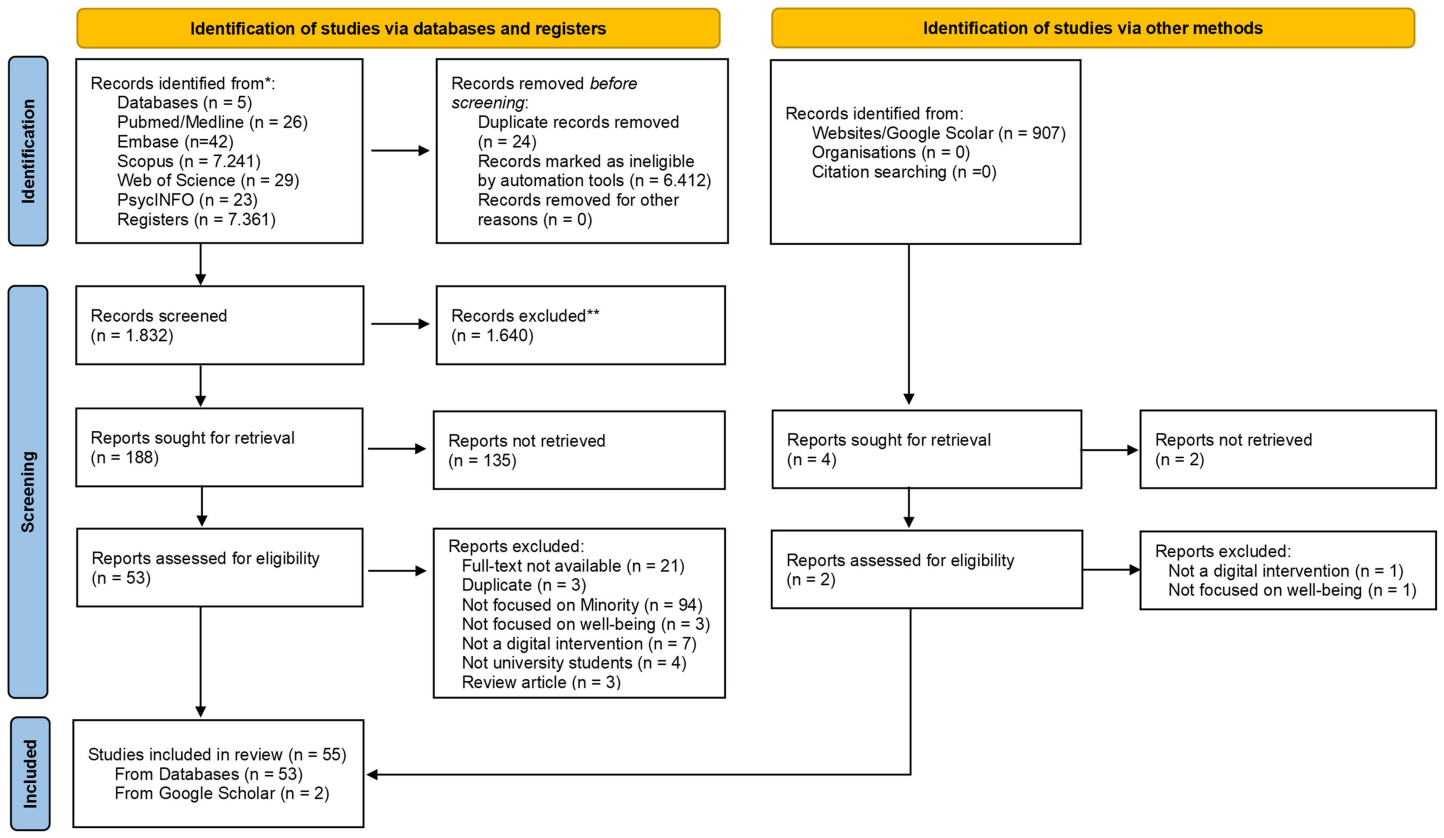
Flowchart of the scoping review based on the PRISMA 2020 model. Source: Adapted from Page et al. (86). Work licensed under CC BY 4.0. To access the license, visit https://creativecommons.org/licenses/by/4.0/. *Records identified from specific databases and registers; **Records excluded by human reviewers or automation tools.

A total of 8,268 records were identified, of which 7,361 came from the PubMed/Medline (*n* = 26), Embase (*n* = 42), Scopus (*n* = 7,241), Web of Science (*n* = 29), and PsycINFO (*n* = 23) databases, in addition to 907 records from the Google Scholar website. After removing 24 duplicates and 6,412 records using automated tools, 1,832 were screened by title and abstract. Of these, 1,640 were excluded because they did not meet the inclusion criteria. In the eligibility stage, 192 texts were retrieved for full reading (188 from the databases and 4 from the website), of which 137 were not retrieved or excluded after evaluation. In the end, 55 studies were included in the review, 53 from the databases and 2 from Google Scholar. The process is described in Figure 1 (PRISMA Flowchart).

A total of 55 empirical studies investigating technological interventions aimed at promoting the wellbeing of university students belonging to social minorities were included in the analysis. The analyzed populations included black, indigenous, immigrant, low-income, and LGBTQIA+ students, with sample sizes ranging from case studies to investigations involving thousands of participants (Table 1).

**Table 1:** Characteristics of included studies on technological interventions in mental health and wellbeing aimed at minority university students.

**Author**	**Year**	**Country**	**Minority and sample (*n*)**	**Technological intervention**	**E-education**	**E-health**	**Study design**	**Study objectives**
Novella et al. (43)	2022	United States	Includes African Americans (49)	Synchronous online counseling intervention (Doxy.me) via video, compared to in-person care.	Educational components of self-reflection, feedback, and development of coping strategies.	Synchronous digital mental health care for anxiety.	Quasi-experimental trial	Compare the effectiveness of online counseling (synchronous video) and in-person counseling using brief solution-focused therapy for anxiety in college students.
Torres et al. (41)	2023	United States	Includes black people (2,834)	Electronic questionnaire.	Academic adaptation assessed using an electronic form.	Symptoms of anxiety, depression, stress, and loneliness were assessed using an electronic form.	Quantitative cross-sectional study	Investigate mental health symptoms and educational factors during the pandemic, focusing on differences by racial/ethnic groups.
MacDonald et al. (44)	2025	United States	Includes immigrants (9)	“Mindfulness-based stress reduction (MBSR) online adaptation” administered via the Zoom videoconferencing platform.	Use of the “Canvas” learning management system.	Focuses on “mindfulness-based interventions (MBIs)” to “promote mental health” and “reduce stress, anxiety, and depression.”	Qualitative (thematic analysis)	Investigate college students' experiences with online MBSR (an administered group intervention) during the COVID-19 pandemic.
Livermon et al. (47)	2025	United States	Includes black people (134)	Digital intervention via mobile app (“Hoos Think Calmly”).	Study/work-related stress in the app.	Reduction of anxiety, assessment of anxiety, depression, and stress symptoms via app.	Mixed methods study	Assess the acceptability, user experience, and suggestions for improvement of a mobile app for reducing anxiety in the university community.
Martins et al (48)	2021	Brazil	Includes black people (Not reported)	Use of alternative digital platforms for data dissemination (observatories, digital newsletters, campaigns, teleconsultations), mobilization through social networks.	Information campaigns via digital media, production and dissemination of booklets, videos, training of community leaders.	Expansion of access to teleconsultations, monitoring of cases through community platforms.	Experience report	Analyze the impacts of COVID-19 on vulnerable communities and popular strategies, including social technologies and solidarity networks.
Quatraro et al. (49)	2024	United States	Includes immigrants (26)	Mindfulness app “Smiling Mind.”	Self-training in mindfulness on the app.	The app measures levels of stress, depression, and self-compassion using validated scales.	Pre-post experimental study	Evaluate the effectiveness of using a mindfulness app to reduce stress and depression and increase self-compassion among nursing students.
Gladstone et al (50)	2021	United States	Includes black women (34)	Online intervention (Willow), via Qualtrics platform.	Modules include mental health education, coping strategies, campus resources.	Digital prevention of depressive and anxious symptoms, reduction of rumination.	Participatory research and pilot study	Adapt and evaluate the acceptability, feasibility, and usefulness of an evidence-based digital intervention (Willow) for depression prevention.
Leventhal et al. (51)	2024	United States	Includes black people (24)	Mobile adaptation of the MindTrails program (“Hoos Think Calmly”).	Suggestions on academic content and university support in app.	Anxiety reduction, with brief sessions (“microdoses”) and cognitive bias modification (CBM-I) techniques using mobile apps.	Qualitative (focus groups)	Adapt and evaluate the acceptability, feasibility, and usefulness of digital intervention (Willow) for the prevention of depression in female university students.
Wang et al. (52)	2024	China	Low income (624)	Health management applications based on artificial intelligence and mHealth.	Digital literacy.	Use of apps for self-management of health (physical and mental monitoring and wellbeing).	Quantitative study with predictive models (UTAUT + Fogg)	Investigate psychosocial, motivational, and technological factors associated with the willingness to use health management apps among female university students.
King et al. (54)	2024	United States	Includes black people (100)	Use of virtual reality (HTC Vive) with the meditation program “ReMind VR: Daily Meditation.”	Not directly presented.	Digital meditation interventions for reducing anxiety symptoms.	Experimental with physiological measurements.	Investigate whether VR and audio meditations reduce physiological arousal (GSR) and anxiety in racial/ethnic minorities.
Kodish et al. (55)	2023	United States	Includes black (35)	Apps, digital programs via smartphone, tablet, laptop, or computer.	Digital guidance, integration with university services, and inclusive outreach materials.	Seeks to increase access, engagement, and adherence to digital mental health interventions.	Modified Delphi	Identify barriers and strategies to increase access and engagement of university students of color in digital mental health interventions.
Conley et al. (79)	2024	United States	Includes immigrants (145)	Mindfulness mobile app (Headspace)	Promotes self-regulation and self-management skills.	Multidimensional assessment: symptoms of depression, anxiety, stress, rumination, and positive wellbeing.	Qualitative cross-sectional study	Evaluate the effects of mindfulness-based digital intervention on psychological distress and wellbeing among university students.
Browning et al. (80)	2020	United States	Includes black people and immigrants (82)	Digital exposure to 360° nature videos through virtual reality (VR).	Educational content on the benefits of restorative nature experiences and the use of immersive technology.	Digital intervention (VR) aimed at promoting mental health and wellbeing (evaluates effects on mood, stress, physiological regeneration, and arousal).	Qualitative exploratory study	Compare the effects of exposure to nature (360° VR, outdoors, indoors) on the mood, restorativeness, and arousal of university students.
Civit et al. (87)	2024	Spain	Includes black people (135)	Software with graphical user interface (GUI) for interaction and videoconferencing software.	Self-regulated learning.	Improving wellbeing, affect balance, and emotion regulation through meditation.	Qualitative case study	Examine the effect of spirituality on self-regulated learning, affect, relationships, and wellbeing among liberal arts college students.
Cao et al. (88)	2022	Thailand	Chinese immigrants (1,022)	Psychological counseling on an online platform.	Adaptation and educational counseling on an online platform	Online psychological counseling with an emphasis on adaptation and coping after the pandemic.	Qualitative study with interviews	Investigate the association between the provision/use of counseling services and the mental health of Chinese international students in Thailand after the pandemic.
Willis (56)	2022	United States	African Americans (38)	Evaluation of existing apps (CBTi, Mood Coach, STAIR, Safe Place).	Psychoeducation on mental health, racial identity, and discrimination, with culturally adapted resources.	Use of apps to reduce symptoms of depression, anxiety, and racial stress	Mixed study (quantitative and qualitative)	Identify desired characteristics in culturally adapted mHealth interventions and assess attitudes of African American youth toward mental health technologies.
Farrer et al (89)	2020	Australia	Immigrants (611)	Uni Virtual Clinic (UVC), a comprehensive online platform with information, screening, self-help tools, and digital therapeutic modules.	Digital educational content on mental health, prevention, symptoms, wellness promotion, and student experiences.	Interpersonal therapy, automated feedback, and mental health referral and support resources.	Development and pilot study	Develop, describe, and evaluate the participatory design of UVC (Uni Virtual Clinic), a scalable online program for college students' mental health.
Theodorou et al. (90)	2023	Italy	Includes non-binary people (113)	Exposure to virtual natural environments (national park, lake environment, and Arctic environment) via virtual reality (VR) with Oculus Quest 2 headset, using 360° panoramic photos.	Application of VR in educational contexts for wellbeing.	Evaluation of VR as subjective vitality and psychological restoration in wellbeing indicators.	Controlled experiment	Investigate the effect of online spiritual classes on emotional regulation and wellbeing in highly skilled university students.
Kodish et al. (39)	2023	United States	University students of color (30)	Online screening (screener) and digital therapy based on iCBT modules.	Personalized feedback via digital screener.	Screening, monitoring, online therapy (CBT).	Mixed study with screening and Delphi	Investigate whether virtual natural environments increase subjective vitality through psychological restoration, comparing three types of virtual nature (park, lake, arctic) with an urban environment.
Rubin et al. (91)	2024	United States	Includes black people (91)	Teleconsultation with mindfulness intervention, using a HIPAA-compliant platform.	Not directly presented.	Measures loneliness, stress, depression, and anxiety via validated scales—UCLA Loneliness, PSS, PHQ-8, GAD-7.	Randomized clinical trial	Evaluate the effectiveness of a single online mindfulness-based intervention (with/without compassion) to reduce loneliness, stress, depression, and anxiety during the pandemic.
Elkhodr et al. (92)	2024	United States	Includes low-income immigrants (not applicable)	Mobile app prototype followed by online treatment allocation (iCBT).	Personalized feedback and treatment guidance.	Self-assessment of symptoms of depression, anxiety, and suicide risk.	Structural and theoretical proposal study	Assess racial/ethnic differences in mental health symptoms and treatment adherence among college students, testing the impact of technology on reducing disparities.
Kodish et al. (58)	2022	United States	Includes black people (2090)	Online cognitive behavioral therapy—iCBT.	iCBT educational modules. Includes personalized feedback and peer guidance.	Online treatment for depression, anxiety, and suicide	Randomized controlled trial	Assess racial/ethnic differences in: (1) severity of mental health symptoms and treatment history; (2) adherence to online/in-person therapy after digital screening.
Mudau et al. (93)	2024	South Africa	Low income (1,100)	Mental health app	Improvement in educational performance through app.	Promotion of mental health through app.	Exploratory study using mixed methods	Explore the feasibility and acceptability of a mental health app intervention among university students.
Feijóo-García et al. (94)	2024	United States	Includes black people (721)	“Virtual humans” (embedded conversational agents)	The educational practice of “gratitude journey”	“Virtual humans” promote wellbeing.	Experimental study	Explore the effects of demographic similarity between user-agent and user-designer in the design of virtual humans to promote mental health intentions in university students.
Macdonald et al. (62)	2022	United States	Includes indigenous and LGBTQ+ people (24)	Centralization and use of online platforms, promotion via social media, and integration of applications/portals for navigation.	Mandatory online courses on stress management, promoting resilience, and inclusion of mental health topics in the curriculum.	Virtual therapies, care, workshops, and group support.	Qualitative study with service providers	Identify facilitators and barriers, from the perspective of providers, to university students' access to mental health services on campus.
Booysen and Slabbert (95)	2025	South Africa	Black people (3)	Prolonged exposure therapy (PE) online, via Zoom.	Online academic support	Treatment of Post-Traumatic Stress Disorder (PTSD) and depression, with online follow-up.	Serial case study	Assess the feasibility and effectiveness of online prolonged exposure therapy for treating PTSD in university students.
Elsom et al. (96)	2021	Australia	Low income (108)	Implementation of an Alternate Reality Game (ARG) during orientation week, using videos, QR codes, and smartphones.	Game designed to promote engagement, social integration, campus exploration, orientation, and skill development.	Verification of student wellbeing through the game.	Mixed methods study with longitudinal data	Assess whether participation in ARG improves engagement, positive emotions, and social integration of students in access programs.
Viskovich and Fowler (97)	2023	Australia	Immigrants (164)	Online intervention based on ACT (Acceptance and Commitment Therapy) called YOLO	Educational components (coping skills, self-awareness, personal values).	Longitudinal online assessment of anxiety, depression, stress, and wellbeing.	Longitudinal study	Assess acceptance and effects of the YOLO program among university students before and during COVID-19, exploring sociodemographic and mental health differences.
Caughlan et al. (98)	2024	United States	Immigrants (387)	Caring Text Messages (CTM)	Not directly presented.	Suicide prevention and mental health support through text messages.	Formative research	Develop and evaluate caring text messages for Native American and Alaska Native youth, college students, and veterans.
Golan et al. (99)	2023	Israel	Includes Arabs (1,438)	Digital learning platform (Learning Management System, LMS)	Academic and personal development content on a digital platform.	Assessment of students' self-esteem, mental health, and wellbeing on a digital platform.	Quasi-experimental with control group	Investigate the mediating effects of self-esteem, body image, and mental health in online courses.
Michikyan (57)	2024	United States	Includes black people (220)	Multidimensional-Identities-Qualitative-Quantitative-Questionnaire (MiQ) electronic questionnaire, digital self-portraits, and electronic narrative profiles.	Digital teaching techniques to value diverse identities and experiences in the classroom.	Addresses aspects of wellbeing, belonging, self-esteem, and engagement.	Qualitative study	Investigate how digital activities value the identities of minority university students and strengthen academic belonging.
Atuahene et al. (63)	2024	China	Black people (879)	Electronic questionnaires administered via Google and WeChat.	Dimension of satisfaction with online teaching.	Perception of psychological safety and institutional support during online teaching.	Cross-sectional study	Investigate how psychological safety, inclusive leadership, institutional support, and student interaction affect African students' satisfaction with online teaching after the pandemic.
Werntz et al. (64)	2023	United States	Includes black people (3141)	Prototype MentorHub app to connect peer mentors with freshmen.	Virtual mentoring.	Health and wellness from an app.	Implementation study	Connect students to university resources through digital mentoring, reducing institutional barriers and improving retention.
Bornschlegl and Caltabiano (100)	2022	Australia	Includes low income (14,578)	Digital platforms such as Studiosity	Digital environments (web, PDF, and online mentoring) for academic support and guidance.	Discussed anxiety in welcoming virtual environments.	Descriptive study	Investigate reasons for seeking or not seeking academic support and how to make it more accessible and engaging for university students.
Williams et al. (65)	2022	United States	Black people (11)	Mental health apps (e.g., Calm) and social media (Instagram, YouTube) as support tools.	Dissemination of mental health information via social media and Black student organizations.	Digital technologies for managing stress and mental health symptoms.	Exploratory qualitative study	Identify the needs and preferences of Black male college students in the use of social media and mobile apps for mental health.
Vega et al. (101)	2023	Indonesia	Low income (80)	Implementation of blended learning with Mobile-Assisted Language Learning (MALL), using Moodle and mobile applications.	Assesses English proficiency, engagement, and virtual participation.	Assesses motivation and academic satisfaction in the virtual environment.	Quasi-experimental	Assess whether blended learning with MALL increases proficiency, motivation, engagement, satisfaction, and perception of accessibility among university students.
Gbollie et al. (102)	2023	South Africa	Includes black people (17,838)	Online therapy (video call), mental health apps, AI chatbots.	Not directly presented.	Wellbeing in intended use.	Cross-sectional survey	Explore attitudes and intention to use digital solutions in mental health among South African university students.
Attridge et al. (103)	2020	United States	Black people and immigrants (951)	Online, self-guided cognitive behavioral therapy (CBT) modules for anxiety, depression, social anxiety, and insomnia; with optional support from a coach (email, text, phone) and “teammate” (friend/family).	Educational content, videos, animations, and CBT exercises, as well as formative assessments, feedback, and homework between lessons.	Digital mental health therapy module to reduce clinical symptoms (anxiety, depression, social anxiety, insomnia) and promote wellbeing.	Longitudinal study	Evaluate the effectiveness, engagement, and moderators of self-guided digital CBT modules in US college students, comparing them with employed adults.
Kazi and Sandbulte (104)	2025	United States	Includes black people (15)	Technological test “FreeMind: Groups”: Flutter/Django application.	Not directly presented.	Stress management via personalized recommendations and online social group support.	Mixed study	Explore how social circles and algorithms in a digital mental health tool support students' stress management.
Dorevitch et al. (105)	2020	Australia	Asian immigrants (624)	Electronic form	Digital assessment of maladaptive perfectionism profiles, focusing on identifying students at risk.	Digital assessment of mental health, including self-esteem and internalized shame, to identify factors associated with depression.	Correlational study	Assess whether maladaptive perfectionism, mediated by self-esteem and shame, predicts depression in different cultural groups of university students.
Fang and Zhu (106)	2022	United States	Contains LGBT people (129,000)	Evaluation of the use and perception of algorithmic matching systems to connect users on online support platforms (OMHC).	Educational peer exchanges on mental health, coping strategies, and self-awareness through peer support.	Emotional support environment, live chats, anonymous connection, 24/7 system, with manual and automated matching.	Exploratory with algorithm prototype	Investigate the challenges, perceptions, and impact of using matching algorithms in forming peer support relationships in online mental health communities.
Hartwig et al. (107)	2024	United States	Black women (6)	Mindfulness-Based Stress Reduction (MBSR) implemented via face-to-face sessions and complementary online activities.	Education on mindfulness and mental health included in a university course.	Promotion of psychological wellbeing and stress reduction, with assessment of multiple outcomes: mental health (anxiety, fatigue, confusion, mood).	Quasi-experimental	Evaluate the effects of a brief MBSR program on black female university students, measuring its impact on psychological, cognitive, and cardiovascular health.
Heron et al. (73)	2019	United States	Includes black people (1,371)	Online survey application to assess mobile technology usage patterns and mHealth messaging preferences for future interventions.	Educational messages for behavior change via mHealth.	Assesses use, access, and preferences for digital health interventions (mental/physical).	Quantitative cross-sectional survey	Assess gender, race, and ethnicity differences in mobile technology use and mHealth messaging preferences among university students to support inclusive digital interventions.
Reyes-Portillo et al. (74)	2022	United States	Includes black people (827)	Behavioral intervention technologies (BITs), with mental health apps (MHAs) and the TAO program.	Improved communication and awareness of technological resources.	Addresses online interventions (TAO) and apps for depression, anxiety, and suicidal ideation	Retrospective database analysis	Investigate attitudes and use of online interventions by at-risk university students (depression, anxiety, suicidal ideation), with a focus on minorities.
Ogundipe et al. (75)	2025	Nigeria	Black people (400)	Digital health platforms (DHP).	Not directly presented.	Perceptions, attitudes, and use of digital health platforms for the wellbeing of university students.	Cross-sectional study with qualitative analysis	Determine the perceptions, attitudes, and use of digital health platforms in promoting mental health among students at a national university in Nigeria.
Oliveira (108)	2022	Brazil	Non-white people (509)	Self-administered online questionnaires (QSD, SRQ-20, QVA-r).	Validated digital instruments for mapping academic experiences.	Digital screening and tracking of mental disorders.	Quantitative cross-sectional study	Analyze the factors associated with common mental disorders in undergraduate students at UFBA, Salvador, 2021.
Feser et al. (66)	2023	Germany	Includes immigrants (238)	Online survey (virtual questionnaire).	Investigation of students' online educational experience.	Completion of an online form on sense of belonging.	Quantitative research	Investigate the sense of belonging among first-year physics students and factors contributing to dropout rates.
Perry et al. (78)	2021	United States	Includes black people (416)	Self-affirmation via digital platform (Qualtrics): students write about important personal values throughout the school year, receiving electronic prompts.	The self-affirmation exercise is educational (encourages reflection on values, self-knowledge, resilience, and coping with stigma/racism).	Use of electronic questionnaires to promote mental health (reduction of fatigue, depression, anxiety; increase in belonging and stability).	Pilot study	Assess whether black students have lower wellbeing, belonging, and goal stability vs. white students, and whether self-affirmation reduces psychosocial disparities.
Liu (109)	2024	China	Immigrants (120)	Use of smart technologies (mobile apps such as Busuu, Lingoda, and LinguaLeo).	Second language learning (English–Chinese) with an emphasis on language skills.	Brain activity and neural connections in apps.	Experimental neuroscientific study	Investigate the impact of bilingualism and the use of smart technologies on brain activity and neural connections in university students.
Benton et al. (110)	2016	United States	Black people and immigrants (1,241)	Therapist-Assisted Online (TAO).	Interactive online educational modules and digital homework via smartphone or tablet.	Weekly therapy videoconferences.	Quasi-experimental	Describe the structure, content, and effectiveness of therapist-assisted online therapy (TAO) for anxiety in a university setting. Compared to face-to-face therapy.
Rodríguez-Rivera et al. (111)	2024	Spain	Includes LGBT people (36)	Cooperative workshop based on the digital game “A Normal Lost Phone” (LGTBI-friendly) on devices (tablets/computers).	Cooperative game-based learning integrated into the subject “Social Skills in Social Education”: groups of 4–6 and group reflections.	Wellbeing and academic satisfaction through gaming.	Quasi-experimental with pre- and post-testing	Assess academic satisfaction, attitudes/beliefs about TGD (TABS), and motivation for cooperative learning (CMELAC) after game intervention.
Lim et al. (112)	2023	Malaysia	Immigrants (214)	Virtual teaching platform.	Challenges and opportunities of virtual platform education.	Personal acceptance, social support, and self-esteem in remote learning.	Quantitative research/cross-sectional survey	Investigate how “mattering,” social support, and self-esteem affect unconditional self-acceptance in remote learning.
Barnwell and Patton (67)	2025	United Kingdom	Immigrants (11)	Digital Mental Health Packages (DMHP): computerized CBT (cCBT) and digital peer support forums, accessed via computer or online application.	Not directly presented.	Online reports on anxiety, depression, stress, loneliness, need for emotional support, anonymous support, and experiences in forums.	Qualitative with reflective thematic analysis	Qualitatively explore university students' experiences with digital mental health packages, assessing benefits, barriers, and recommendations.
Hendrickse et al. (68)	2024	United States	Includes non-white individuals (2,016)	Interactive website “Student Resilience Project”: multimedia content (instructional videos, skill-building, TED-style podcasts).	Digital educational content focused on developing coping skills (yoga, journaling, breathing techniques) and videos.	Online mental health and resilience promotion intervention aimed at reducing stress and strengthening self-efficacy to seek help.	Implementation study	Assess whether high exposure to the website increases self-efficacy for stress and self-help intentions in college students, verifying effects on non-whites and sexual minorities.
Naidoo and Cartwright (69)	2022	South Africa	Black people (not applicable)	Discussion on the adoption of telemental health.	Describes emergency transition to e-learning and challenges in accessing digital resources.	Emphasis on expanding the reach of psychological support services through digital means (remote counseling, videos, podcasts, hotlines, self-help).	Theoretical/reflective essay.	Analyze the impact of the pandemic on mental health, psychosocial vulnerability, and access to psychological support services for university students.

The technological interventions consisted of various applications of digital technologies in Digital EduHealth actions. Many tools were recurrent, such as mobile applications, online platforms, virtual reality, chatbots, social networks, and virtual learning environments, but their use varied according to purpose: educational, assistance, or both. In e-education, technologies were used in strategies such as online courses, digital games, self-affirmation activities, self-regulated learning, digital literacy, and inclusive teaching materials. In e-health, these technologies were used for psychosocial care via mental health apps, digital screenings, online therapy, digital mindfulness, remote counseling, therapeutic virtual reality, and virtual humans. Often, the same intervention operated in both fields simultaneously, reinforcing the integrated nature of Digital EduHealth.

The studies employed various methodologies and largely demonstrated the positive effects of digital interventions. Results showed alleviation of symptoms such as anxiety, depression, stress, and loneliness. Furthermore, the interventions promoted emotional self-regulation, coping mechanisms, and a sense of academic belonging. Those with personalized support and cultural sensitivity were the most effective. However, barriers such as digital exclusion, institutional racism, and the invisibility of marginalized identities were also evident. In response, good practices based on co-creation, digital inclusion, and the valorization of intersectionalities emerged. Emerging technologies such as virtual reality, artificial intelligence, and digital agents proved especially promising in highly vulnerable contexts, particularly during the pandemic.

**Figure 2:**
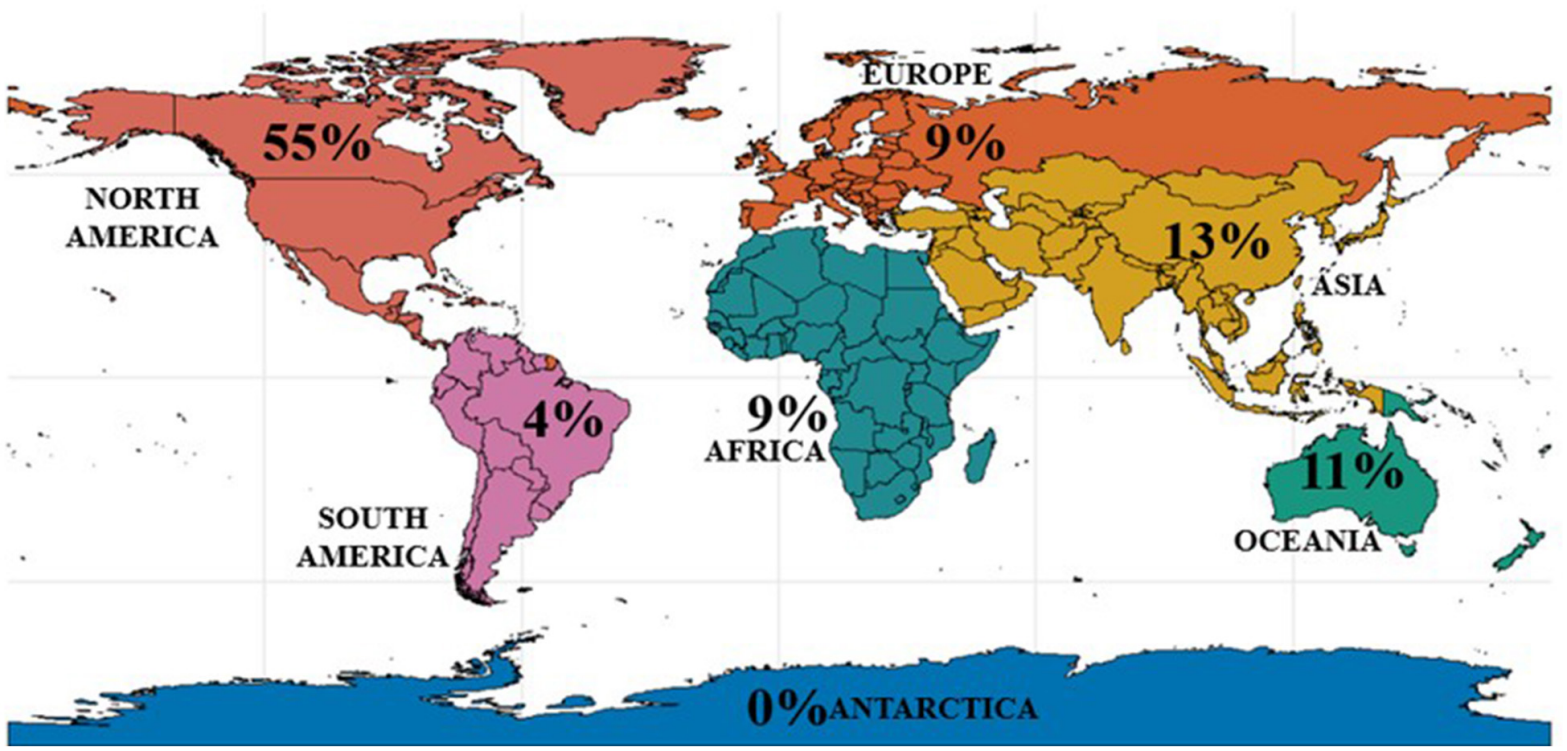
Global map of studies on Digital EduHealth interventions aimed at the wellbeing of minority university students, by continent (*N* = 55).

The geographic distribution of studies on Digital EduHealth interventions shows a significant concentration in North America, accounting for 55% (n = 30) of the included publications. Asia followed with 13% (*n* = 7), then Oceania with 11% (*n* = 6). Africa and Europe both had 9% (*n* = 5). South America had the lowest representation with only 4% (*n* = 2) of the studies. No publications focusing on Antarctica were found (Figure 2).

Of the 55 studies analyzed, the effects of Digital EduHealth interventions aimed at the wellbeing of university students from social minorities fell into four main categories. The most frequent category was emotional health, with 24 studies reporting a reduction in symptoms such as anxiety, stress, depression, and psychological distress. These studies also reported improvements in emotional self-regulation, self-esteem, subjective vitality, general wellbeing, and resilience. The second most significant category, present in 19 studies, was recognition and engagement. This category highlighted increased acceptance and use of digital interventions, cultural identification with content, improved empathic listening, perception of support, appreciation of diversity, and overcoming institutional and social barriers.

**Figure 3:**
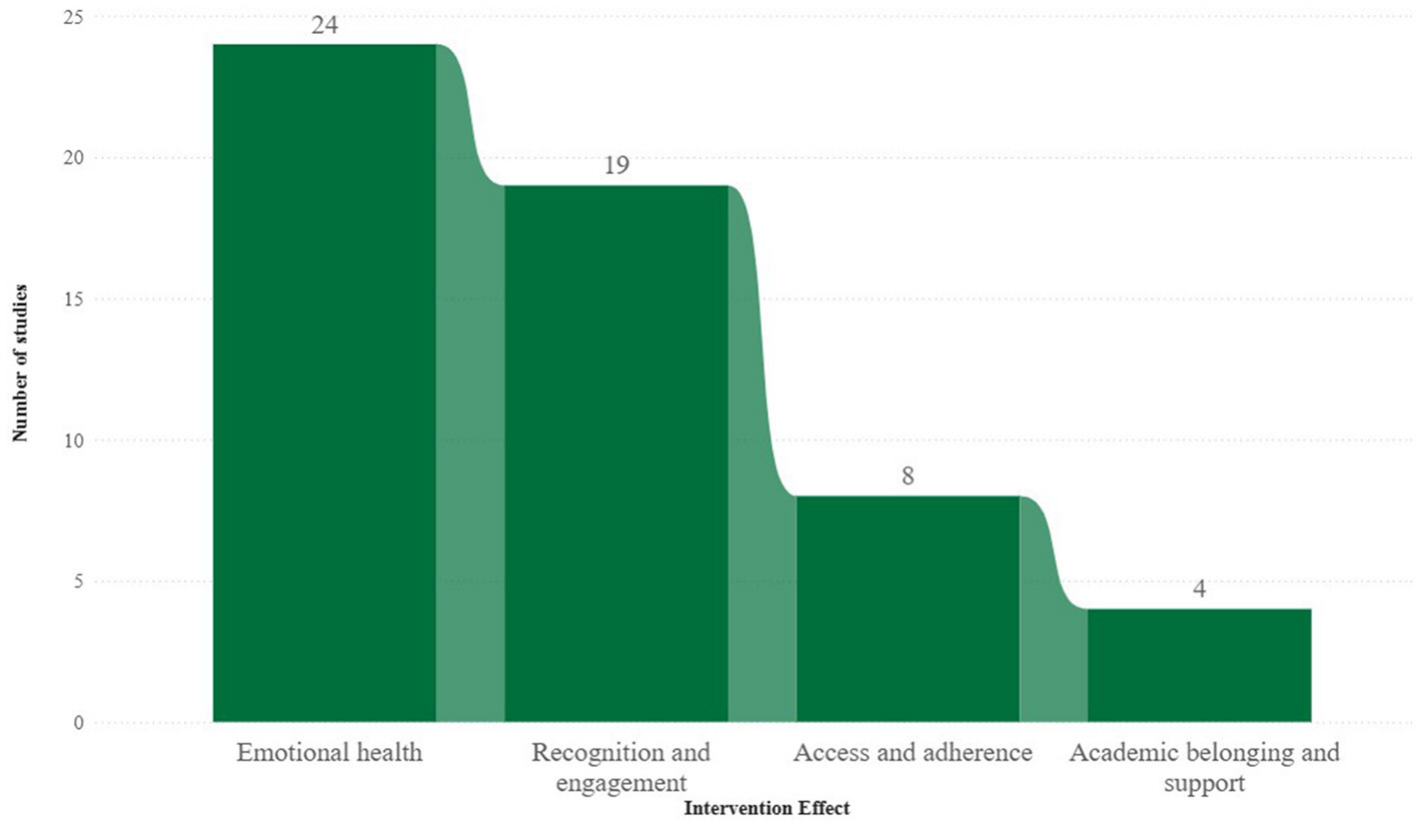
Reported effects of Digital EduHealth interventions aimed at the wellbeing of university students belonging to social minorities (*N* = 55).

Eight studies covered the category of access and adherence, which encompasses effects related to expanding access to technologies, reducing technological and institutional barriers, and increasing the intention or continuity of use of digital tools. Finally, the category of academic belonging and support was identified in only four studies and involved positive impacts on strengthening academic ties, promoting student identity and satisfaction with learning, and expanding support for remaining in higher education (Figure 3).

**Figure 4:**
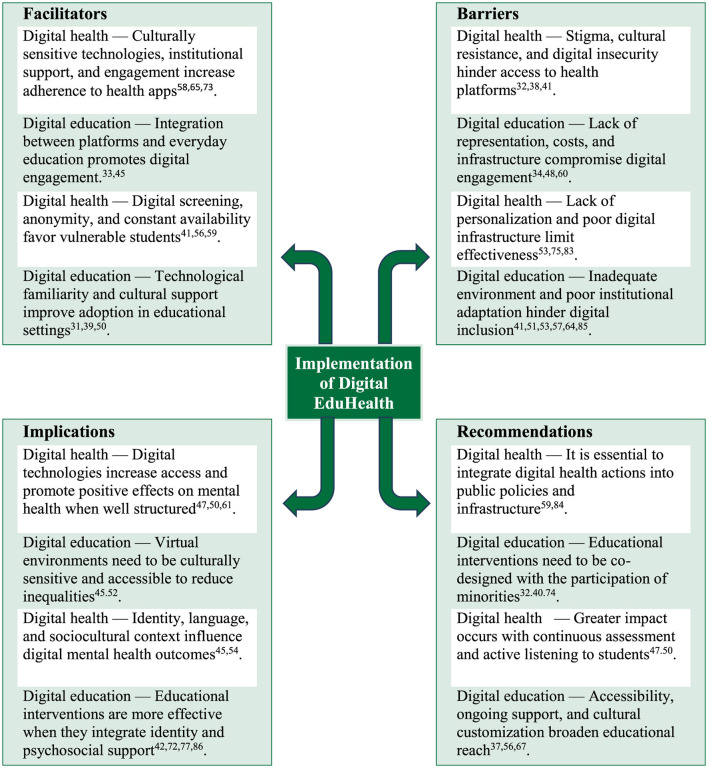
Factors associated with the implementation of Digital EduHealth interventions to promote the wellbeing of university students from social minorities.

Figure 4 illustrates the factors associated with implementing Digital EduHealth interventions that promote the wellbeing of university students from social minorities. The use of culturally sensitive technologies, institutional support, integration with everyday educational life, technological familiarity, and continuous digital resource availability are among the facilitators that favor engagement, especially among vulnerable students.

However, implementation faces barriers such as stigma, cultural resistance, digital insecurity, a lack of personalization and representation, high costs, poor infrastructure, and poorly adapted institutional environments. These barriers compromise adherence, effectiveness, and inclusion.

Observed implications indicate that well-structured digital interventions can expand access and positively impact student wellbeing, particularly when considering identity, language, and sociocultural context. Integrating psychosocial support and cultural sensitivity into virtual environments is also essential to reducing inequalities and increasing effectiveness.

We highlight the following recommendations: integrate digital actions into public policies and existing infrastructure; adopt co-design strategies with the active participation of social minority groups; ensure continuous evaluation with active student feedback; and expand accessibility, support, and cultural personalization to increase the reach and impact of interventions.

## Discussion

This scoping review identified 8,268 records. After applying the PRISMA 2020 model, 55 empirical studies were included in the final analysis. These studies examined technological interventions that promote the wellbeing of university students belonging to social minorities and historically marginalized groups. The significant reduction in the initial sample size indicates the scarcity of research on this population and the low scientific and political prioritization of the topic from the outset.

The analyzed interventions employed various digital technologies in the fields of e-education (digital education) and e-health (digital health), confirming the convergence of pedagogical and psychosocial strategies within the scope of Digital EduHealth. In the educational field, online courses, self-affirmation, games, digital literacy, and inclusive pedagogical environments were notable. In care approaches, practices such as online therapy, digital screenings, mindfulness, remote counseling, therapeutic virtual reality, and digital agents were identified. Often, technologies operated in both dimensions simultaneously, reinforcing the potential synergy between emotional support and academic development.

Methodologically, the studies ranged from descriptive designs to randomized trials with varied samples and positive results. The interventions were effective at reducing symptoms such as anxiety, stress, depression, and loneliness, and at promoting emotional self-regulation, self-esteem, and academic belonging. However, the lowest occurrence was observed in direct academic impact, revealing a gap in interventions that explicitly strengthen university retention.

Geographically, more than half of the studies were conducted in North America. There was less participation from Asia, Oceania, Africa, Europe, and South America, respectively. This distribution indicates a significant imbalance in the development and validation of technologies for student populations from social minorities, with limited representation from countries in the Global South. Many of the structural inequalities affecting these students—many of whom benefit from affirmative action—are especially critical in these contexts. Thus, the predominance of research in Northern countries highlights a bias in the origin and application of the analyzed technologies, with limited attention to the cultural and institutional diversity of global education systems.

This scenario contrasts with ongoing efforts in countries such as South Africa, which articulates e-Health and e-Education as equity strategies (9, 19). In the European Union, Regulation (EU) 2021/522 emphasizes the importance of interoperability and citizen participation in digital transformation (20). In Brazil, the Ministry of Health launched the Digital Health Strategy for Brazil 2020–2028 (ESD 28) in 2020, aligning with WHO guidelines and continuing the guidelines previously established by the National Policy on Health Information and Informatics (PNIIS). ESD 28 focuses on health service users and enhancing information, services, and process support to improve care (31). Notable initiatives include Conecte SUS (21), now SUS Digital (22), and the integration of digital practices in education (32), which have gained momentum through the recent PET-Saúde Digital call for proposals (33), promoting shared governance between universities and municipal health departments. These actions reveal concrete paths for inclusive digital transformation in the Global South and can inspire similar strategies in other international contexts.

This mismatch, marked by the predominance of studies concentrated in the Global North, is made more paradoxical by the fact that affirmative action and/or university retention policies are being implemented or discussed not only in countries such as the United States, Canada, France, Finland, Israel, and Australia, but also in Brazil, Colombia, Ecuador, Honduras, Uruguay, India, South Africa, China, Malaysia, Pakistan, Sri Lanka, Nigeria, Romania, Hungary, Greece, Indonesia, and Nepal (34–38).

The analysis identified factors that facilitate or hinder the implementation of these technologies. The cultural sensitivity of the interventions, institutional support, integration into everyday educational life, familiarity with digital resources, and continuous availability of tools are among the facilitators that stand out. These conditions enhance adherence and impact among the most vulnerable students. Conversely, barriers include stigma, cost, cultural resistance, digital insecurity, lack of representation, poor infrastructure, and lack of support policies. These factors compromise the effectiveness and scalability of these interventions.

Despite the observed advances, none of the 55 included studies evaluated technological interventions specifically aimed at students benefiting from affirmative action. Nor did they explicitly discuss health or digital education policies aimed at university retention. This is a significant gap, especially in countries such as Brazil, where racial, social, and educational inequality are intertwined with a history of institutional exclusion. The absence of approaches anchored in the experiences of students who benefit from affirmative action not only undermines the effectiveness of the developed technologies but also perpetuates the invisibility of a target audience fundamental to advancing equity in higher education.

The findings of this review directly impact the promotion of equity in higher education and the development of socioculturally sensitive health technologies. The evidence suggests that digital interventions that focus on emotional health, identity recognition, and academic support are most effective when they incorporate strategies such as empathic listening, cultural personalization, and remote psychopedagogical support. These elements encourage the participation of students from minority groups, particularly black, indigenous, LGBTQIA+, immigrant, and low-income students.

Students from social minorities face institutional, emotional, and cultural barriers that increase their vulnerability to psychological distress and dropping out of college. Recent evidence shows that digital interventions effectively reduce symptoms of anxiety and depression, particularly when they incorporate human support. The results of this review indicate that actions integrating cultural sensitivity, empathetic listening, and continuous digital support can function as protective measures, promoting emotional self-regulation, a sense of belonging, and identity recognition (13, 39).

Failing to consider the unique characteristics of quota students reproduces the logic of invisibility that marks their institutional trajectory. The lack of integration between public affirmative action policies and technological solutions undermines the emancipatory potential of Digital EduHealth.

From a practical point of view, the findings point to the need for intersectoral strategies in which higher education institutions, public managers, and technology developers collaborate. Systemic approaches, as proposed by the Good Things Foundation (40) and the Association of Research Libraries (17), should ensure infrastructure, digital literacy, accessibility, and ongoing emotional support. These strategies promote engagement and are fundamental to transforming historically exclusionary educational environments into more diverse, inclusive, and equitable spaces.

This review shows that the effectiveness of Digital EduHealth interventions depends on their sensitivity to the intersectionalities that comprise the student experience, particularly when it is shaped by historical exclusion based on race, class, gender, and other social factors (41). Highlighting this aspect is an improvement over previous studies (42), which emphasized the importance of tele-education and telehealth in addressing educational disparities during the pandemic. While these studies recognized important sociocultural aspects, they were limited in their consideration of cultural adaptations of technologies for minority populations and multiple intersectionalities, as demonstrated by more recent findings (43, 44).

Studies in the United States reveal systematic inequalities in subjects such as chemistry and physics. Black, Latino, and low-income students tend to perform worse academically, even in contexts with support strategies like tutors and learning assistants (45, 46). These inequalities are seen as reflections of “society's educational debts,” stemming from centuries of exclusion and disinvestment. However, these approaches rarely consider the potential of digital technologies to mediate these asymmetries. The finds of this review partially fill that gap (47–52).

Students who attach greater importance to their racial identity tend to support affirmative action more. Students who attended historically black institutions expressed less support than those who attended predominantly white institutions. This suggests that institutional context influences these attitudes (53). Although the study focused more on attitudes than on adherence to interventions or aspects of resilience, the findings reinforce the relevance of the racialized context—understood as the presence of structures, norms, and experiences marked by race relations—in the university experience of black students and other social minorities. This influence is corroborated by studies investigating the physiological responses of racial and ethnic minorities to digital interventions, barriers, and strategies for engaging students of color in digital mental healthcare, as well as by research on identities and sense of belonging among minority young adults (54–57).

In this scenario, Digital EduHealth proves to be an ally in strengthening identity and addressing institutional loneliness among minority students. The most effective interventions, beyond clinical or pedagogical objectives, are those guided by cognitive justice and intersectional welcoming, as demonstrated by studies on racial equity in digital health and technology-mediated strategies for academic belonging (57, 58).

In Brazil, despite the advances promoted by quota policies, challenges to student retention persist, especially due to a lack of emotional, financial, and institutional support (59–61). This review points out that, when developed with inclusive intent, digital solutions can mitigate these barriers. Evidence of this comes from studies on social media support, digital wellness interventions, and changes in counseling services during the pandemic (62–69).

Students exposed to institutional racism experience greater negative impacts on their mental health, especially in the absence of culturally sensitive support (70, 71). Institutional coping strategies still lack greater reach and visibility (72). These findings align with what this review demonstrates: the effectiveness of digital interventions is directly proportional to their ability to adapt to the specific experiences and needs of racial and ethnic groups (73–75). In this sense, technological access must be accompanied by infrastructure, digital literacy, and psychosocial support to reduce inequalities (76).

Afro-diasporic intellectuals recognize the historical exclusion of black populations from access to health and education as a form of symbolic genocide (77), which reinforces the urgent need for technologies committed to social justice. When developed by diverse teams attentive to structural inequalities, Digital EduHealth can take on a remedial role to promote belonging and identity among black students and those from social minorities (78). Evidence from this review also shows that digital interventions with this potential align with strategies based on mindfulness and immersive experiences with nature (79, 80) as well as other educational technologies focused on emotional and relational wellbeing. The international literature emphasizes the importance of creating sustainable digital ecosystems that consider technological sovereignty, data protection, and equitable access (81), including in contexts of extreme exclusion, such as prisons. There, hybrid pedagogical technologies have been shown to promote citizenship and wellbeing (82).

It is important to note that mental health support offered by digital technologies does not replace direct human contact, especially for young people who are overexposed to screens. While universities should invest in technology-based wellbeing programs, they must also ensure the provision of conventional therapies, therapeutic groups, and other forms of support within the university environment.

## Conclusion

This scoping review identified structural barriers and emerging opportunities regarding the use of digital technologies for the wellbeing and mental health of university students from historically excluded groups. Despite the thematic and geographic diversity of the studies analyzed, there was a lack of digital solutions specifically designed for quota students. This limits the effectiveness of interventions in addressing racial and socioeconomic inequalities in higher education.

Despite its scope, this review has significant limitations. The scarcity of studies focused exclusively on university students from social minorities required the inclusion of broader research in which these groups appear only secondarily. This limitation compromises more specific analyses, especially regarding beneficiaries of affirmative action, a dimension almost never mentioned in the studies included in this review. This is surprising, considering this population symbolizes affirmative action and retention policies worldwide. Additionally, the fragmentation between health and education in intervention design hindered the integrated evaluation of Digital EduHealth as an academic wellbeing policy. There were also difficulties in clearly defining the methodological designs, which required the team to make inferences that may have introduced classification bias. The absence of standardized indicators of mental health and academic engagement further complicated the task of comparing studies. Finally, the lack of specific analyses of technological interventions for the wellbeing of quota students reduced the potential of the review to inform inclusive retention policies based on digital technologies.

There is an urgent need to develop digital interventions targeting students who benefit from affirmative action, particularly in developing countries where structural inequalities directly impact racial and socioeconomic factors, thereby undermining the effectiveness of higher education retention policies. These interventions should be co-created with students, incorporating critical digital literacy, active listening, and academic retention support. Additionally, future research must adopt indicators sensitive to minority experiences, considering mental health, institutional climate, racism, and belonging. Using standardized digital transformation frameworks, such as those proposed by the Pan American Health Organization (PAHO) (83) and other international agencies of the European Union (84) and Asia-Pacific (45, 85) can help qualify the evidence and allow for more effective comparisons between contexts, especially in terms of data interoperability.

By highlighting these gaps, the review provides concrete support for developing more inclusive, intersectional technologies aligned with retention policies. The evidence gathered also strengthens the argument that integration between the fields of health and education is essential to repositioning Digital EduHealth as a public strategy for equity and social justice.

## Data Availability

The original contributions presented in the study are included in the article/Supplementary material, further inquiries can be directed to the corresponding author.

## Author's note

We thank JL for his methodological contributions and technical support throughout this review. We also thank the JN, CM, and MN for making this exchange possible. We thank SD-T for her hospitality and guidance during the research process.
