# Supplementary material for: Digital EduHealth for the wellbeing of minority university students: a scoping review
Source: Front Public Health. 2026 Mar 20;14:1781600. doi: 10.3389/fpubh.2026.1781600 (PMC13048269; doi:10.3389/fpubh.2026.1781600)
Supplement: Supplementary file 1 [file Data_Sheet_1.pdf]

Supplementary Appendix

Digital EduHealth for the Well-Being of Minority University Students: A Scoping Review

The research team prepared this supplementary material to provide readers with additional, detailed information about the development of the research project.

Summary

1 List of acronyms 3
2 Definitions and digital technologies in Digital EduHealth 4
3 PRISMA Checklist 8
4 Form (Rayyan) 10
5 Excluded studies and reasons 11
6 Complete search strategy 22
6.1 PubMed/MEDLINE 22
6.2 Embase 22
6.3 Scopus 23
6.4 Web of Science 24
6.5 PsycINFO 24
6.6 Google Scholar 25
7 Descriptors, keywords, and "single" Boolean logic 27

1 List of Acronyms

Acronym	Meaning
**WHO**	World Health Organization
**PAHO**	Pan American Health Organization
**UN**	United Nations
**IBGE**	Brazilian Institute of Geography and Statistics
**UFBA**	Federal University of Bahia
**CAPES**	Coordination for the Improvement of Higher Education Personnel
**JBI**	Joanna Briggs Institute
**PRISMA-ScR**	Preferred Reporting Items for Systematic Reviews and Meta-Analyses extension for Scoping Reviews
**PCC**	Population, Concept, Context (Framework)
**OSF**	Open Science Framework
**BI**	Business Intelligence
**SUS**	Unified Health System

Supplementary Table 1 - List of acronyms

2 Definitions and Digital Technologies in Digital EduHealth

Term/Technology	Definition	Reference
**Digital EduHealth**	A subset of digital health technologies that integrates educational components to promote the psychosocial well-being of university students, encompassing interventions, digital skills development, equitable access to technological tools, and the construction of psychosocial and educational support environments mediated by digital solutions.	European Union (2020); WHO (2021); PAHO (2021)
**Digital Health**	Application of digital tools in health, involving information technology, management, innovation, and care mediated by digital technologies, including big data, telecare, and interoperability.	Eysenbach (2001); WHO (2021)
**Digital Education**	Integration of digital technologies to expand access, improve the quality of learning, and develop digital skills for living and working in technologized societies.	European Union (2020)
**Digital Technologies**	A set of tools, devices, and electronic infrastructures that enable the collection, processing, communication, and storage of data in virtual or hybrid environments.	Selwyn (2021)
**Mental Health**	A state of well-being in which an individual realizes their potential, copes with the stresses of life – including daily challenges, trauma, loss, grief, abuse, crises, and dependencies, among others – and contributes to their community, while working productively and fruitfully.	WHO (2018)
**Well-being**	A multidimensional condition that encompasses life satisfaction, positive functioning, emotional balance, and existential meaning.	Dodge et al. (2012)
**Affirmative Action**	Policies and practices designed to correct historical inequalities and promote equity, especially in education and employment for underrepresented groups.	Htun (2004)
**Structural Racism**	Racism rooted in social, political, and institutional structures, perpetuating inequalities and exclusions even without deliberate individual actions.	Almeida (2019); Collins & Bilge (2016)
**University Students**	Individuals enrolled in higher education institutions, experiencing academic, social, and psychosocial challenges specific to the university environment.	Tinto (1993); Silva (2020)

**Digital Mental Health Interventions**	A set of actions that use digital technologies (apps, digital data, social media, chatbots, VR, wearables, telepsychiatry, etc.) for mental health promotion, prevention, and treatment.	Torous et al. (2021)

Continued...

**Term/Technology**	**Definition**	**Reference**
**Mental health apps**	Digital applications that offer evidence-based psychological interventions for self-management of symptoms (e.g., CBT, mindfulness, etc.).	Torous et al., 2021, p. 319, 323, 329
**Active data collection**	Digital questionnaires (surveys, EMA) conducted via smartphone to monitor symptoms, mood, and cognition in real time.	Torous et al., 2021, p. 320
**Passive data collection / Digital phenotyping**	Data collected automatically by smartphone/wearable sensors (e.g., GPS, accelerometer), capturing activity and behavior patterns.	Torous et al., 2021, p. 320
**Machine learning and AI applied to digital data**	Algorithms that analyze large volumes of digital data for risk prediction, personalized interventions, and digital biomarkers in mental health.	Torous et al., 2021, p. 320, 329
**Social media**	Digital social media platforms, such as Facebook, Twitter, Instagram, and Pinterest, which can be used both for large-scale monitoring of mood and anxiety through automated analysis of posts (NLP) and for early detection of symptoms, promotion of peer support, and implementation of moderate therapeutic interventions in mental health.	Torous et al., 2021, p. 321, 329
**Chatbots**	Automated conversation systems for screening, emotional support, psychoeducation, and referral, via text, voice, or avatar.	Torous et al., 2021, pp. 321-322, 329
**Virtual reality (VR) and immersive realities**	Simulated environments for controlled exposure, social skills training, mindfulness, and exposure-based therapy, especially for anxiety/PTSD.	Torous et al., 2021, p. 322, 329
**Online self-management platforms (iCBT, self-help)**	Digital platforms with structured cognitive behavioral therapy and self-help modules for self-management of symptoms, generally for young people/adults.	Torous et al., 2021, pp. 326-327
**Wearables**	Wearable devices that collect physiological data (sleep, activity, heart rate), integrating the information into mental health monitoring.	Torous et al., 2021, p. 320
**Telepsychiatry**	Consultations and clinical follow-up conducted via videoconferencing, expanding access to psychiatric and psychological care.	Torous et al., 2021, p. 318, 329

**Interoperability of digital data in mental health**	Ability to integrate, share, and validate digital data (active and passive from apps, wearables, sensors, and platforms) between different systems and professionals using open standards (e.g., SMART on FHIR).	Torous et al., 2021, p. 329

Supplementary Table 2 - Definitions and Digital Technologies in Mental Health

References

Almeida, S. Racismo estrutural. São Paulo: Pólen, 2019.

Collins, P. H.; Bilge, S. Intersectionality. Cambridge: Polity Press, 2016.

Dodge, R.; Daly, A. P.; Huyton, J.; Sanders, L. D. The challenge of defining wellbeing. International Journal of Wellbeing, v. 2, n. 3, p. 222-235, 2012. Available at: https://www.internationaljournalofwellbeing.org/index.php/ijow/article/view/89. Accessed on: May 28, 2025.

Eysenbach, G. What is e-health? Journal of Medical Internet Research, v. 3, n. 2, e20, 2001. Available at: http://www.jmir.org/2001/2/e20/. Accessed on: May 28, 2025.

Htun, M. From “Racial Democracy” to Affirmative Action: changing state policy on race in Brazil. Latin American Research Review, v. 39, n. 1, p. 60-89, 2004. Available at: https://www.jstor.org/stable/1555637. Accessed on: May 28, 2025.

OPAS – Organização Pan-Americana da Saúde. Introdução à Interoperabilidade Semântica | Kit de Ferramentas de Transformação Digital. Washington, D.C.: OPAS, 2021. Available at: https://www.paho.org/pt/documentos/introducao-interoperabilidade-semantica-kit-ferramentas-transformacao-digital. Accessed on: May 28, 2025.

Selwyn, N. Education and Technology: key issues and debates. 3. ed. London: Bloomsbury Academic, 2021.

Silva, S. A. Juventudes e universidade. Salvador: Ed. UFBA, 2020.

Tinto, V. Leaving college: rethinking the causes and cures of student attrition. 2. ed. Chicago: University of Chicago Press, 1993.

Torous, J.; Bucci, S.; Bell, I. H.; et al. The growing field of digital psychiatry: current evidence and the future of apps, social media, chatbots, and virtual reality.

Psychiatry, v. 20, n. 3, p. 318-335, 2021. Available at: https://www.ncbi.nlm.nih.gov/pmc/articles/PMC8423594/. Accessed on: May 28, 2025.

União Europeia. Plano de Ação para a Educação Digital 2021-2027. Bruxelas: União Europeia, 2020. Available at: https://education.ec.europa.eu/pt-pt/focus-topics/digital-education/action-plan. Accessed on: May 28, 2025.

World Health Organization (WHO). Mental health: strengthening our response. Fact Sheet N°220. Geneva: WHO, 2018. Available at: https://www.who.int/news-room/fact-sheets/detail/mental-health-strengthening-our-response. Accessed on: May 28, 2025.

World Health Organization (WHO). Global strategy on digital health 2020-2025. Geneva: World Health Organization, 2021. Available at: https://apps.who.int/iris/handle/10665/344249. Accessed on: May 28, 2025.

3 PRISMA Checklist

Preferred Reporting Items for Systematic Reviews and Meta-Analyses Extension for Scoping Reviews (PRISMA-ScR) Checklist

SECTION	ITEM	PRISMA-ScR CHECKLIST ITEM	REPORTED ON PAGE #
**TITLE**			
Title	1	Identify the report as a scoping review.	1
**ABSTRACT**			
Structured summary	2	Provide a structured summary that includes (as applicable): background, objectives, eligibility criteria, sources of evidence, charting methods, results, and conclusions relating to the review questions and objectives.	1
**INTRODUCTION**			
Rationale	3	Describe the rationale for the review in the context of what is already known. Explain why the review questions and objectives lend themselves to a scoping review approach.	2-3
Objectives	4	Provide an explicit statement of the questions and objectives being addressed with reference to their key elements (e.g., population or participants, concepts, and context) or other relevant key elements used to conceptualize the review questions and/or objectives.	3
**METHODS**			
Protocol and registration	5	Indicate whether a review protocol exists; state if and where it can be accessed (e.g., a Web address); and if available, provide registration information, including the registration number.	4
Eligibility criteria	6	Specify characteristics of the sources of evidence used as eligibility criteria (e.g., years considered, language, and publication status), and provide a rationale.	4-5
Information sources*	7	Describe all information sources used in the search (e.g., databases with dates of coverage and contact with authors to identify additional sources), as well as the date the most recent search was executed.	4
Search	8	Present the full electronic search strategy for at least one database, including any limits used, such that it can be repeated.	Appendix
Selection of sources of evidence†	9	Describe the process used to select sources of evidence for the scoping review (i.e., screening and eligibility).	5
Data charting process‡	10	Describe the methods used to chart data from the included sources of evidence (e.g., calibrated forms or forms tested by the team before use, and whether data charting was done independently or in duplicate) and any processes used to obtain and confirm data from investigators.	5
Data items	11	List and define all variables for which data were sought and any assumptions and simplifications made.	5 and appendix
Critical appraisal of individual sources of evidence§	12	If applicable, provide a rationale for conducting a critical appraisal of the included sources of evidence and describe the methods used, as	-

SECTION	ITEM	PRISMA-ScR CHECKLIST ITEM	REPORTED ON PAGE #
		well as how this information was used in any data synthesis (if appropriate).	
Synthesis of results	13	Describe the methods used to handle and summarize the charted data.	5
**RESULTS**			
Selection of sources of evidence	14	Present the number of sources of evidence screened, assessed for eligibility, and included in the review, with reasons for exclusions at each stage, ideally using a flow diagram.	5
Characteristics of sources of evidence	15	For each source of evidence, present the characteristics for which data were charted and provide the citations.	6-11
Critical appraisal within sources of evidence	16	If applicable, present data on the critical appraisal of the included sources of evidence (see item 12).	-
Results of individual sources of evidence	17	For each included source of evidence, present the relevant charted data relating to the review questions and objectives.	6-11
Synthesis of results	18	Summarize and/or present the charting results as they relate to the review questions and objectives.	12-14
**DISCUSSION**			
Summary of evidence	19	Summarize the main results (including an overview of concepts, themes, and types of evidence available), link the results to the review questions and objectives, and consider their relevance to key groups.	15-16
Limitations	20	Discuss the limitations of the scoping review process.	18
Conclusions	21	Provide a general interpretation of the results with respect to the review questions and objectives, as well as the potential implications and/or next steps.	18
**FUNDING**			
Funding	22	Describe the sources of funding for the included sources of evidence, as well as the sources of funding for the scoping review. Describe the role of the scoping review funders.	19

JBI = Joanna Briggs Institute; PRISMA-ScR = Preferred Reporting Items for Systematic Reviews and Meta-Analyses Extension for Scoping Reviews.
* Where *sources of evidence* (see second footnote) are compiled from, such as bibliographic databases, social media platforms, and websites.
† A more inclusive/heterogeneous term used to account for the different types of evidence or data sources (e.g., quantitative and/or qualitative research, expert opinion, and policy documents) that may be eligible in a scoping review, as opposed to only studies. This is not to be confused with *information sources* (see first footnote).
‡ The frameworks by Arksey and O'Malley (6) and Levac et al. (7) and the JBI guidance (4, 5) refer to the process of data extraction in a scoping review as "data charting."
§ The process of systematically examining research evidence to assess its validity, results, and relevance before using it to inform a decision. This term is used for items 12 and 19 instead of "risk of bias" (which is more applicable to systematic reviews of interventions) to include and acknowledge the various sources of evidence that may be used in a scoping review (e.g., quantitative and/or qualitative research, expert opinion, and policy document).

From: Tricco AC, Lillie E, Zarin W, O'Brien KK, Colquhoun H, Levac D, et al. PRISMA Extension for Scoping Reviews (PRISMAScR): Checklist and Explanation. Ann Intern Med. 2018;169:467–473. doi: 10.7326/M18-0850.

4 Form (Rayyan)

Item	Details / Comments
**Data source**	The data were initially extracted from the Rayyan platform after screening the included articles.
**Subsequent refinement**	Additional variables were included in the form to deepen the analysis, considering the specific objectives of the review.
**Fields included**	The following are the final fields used in the extraction and categorization spreadsheet:
**Key**	Unique study identifier (may be the name of the first author and year).
**Title**	Full title of the study.
**Include**	Indication of whether the study was included or not (yes/no).
**Reason**	Justification for inclusion or exclusion of the study.
**Author**	Name of the author(s).
**Year**	Year of publication.
**Country**	Country where the study was conducted.
**Continent**	Corresponding continent.
**Population**	Description of the population studied.
**E-education**	Indication of integrated digital educational elements (e.g., virtual environments, remote teaching, etc.).
**E-Health**	Digital health elements (e.g., mHealth, telecare, self-care platforms, etc.).
**Objective**	Objective of the study as described by the authors.
**Journal**	Name of the journal.
**ISSN**	ISSN number of the journal.
**Volume**	Volume of the journal.
**Issue**	Issue number.
**Authors**	Complete list of authors.
**Language**	Language of publication.
**Publisher**	Name of the publisher.
**Location**	Location of the institution or context of the study.
**Abstract**	Summary of the study.
**Notes**	Additional notes made by the team.
**DOI**	Digital Object Identifier.
**Keywords**	Keywords provided by the authors.
**pubmed_id**	PubMed identifier, if available.
**pmc_id**	PubMed Central identifier, if applicable.

Supplementary Table 3 - Rayyan Form

5 Excluded studies and reasons

N	Key	Author	Title	Reason
**1**	**rayyan-250332702**	Bernadska et al	"I Feel Disconnected … but Do I?": University Students' Sense of Belonging in Time of Adversity	Article not available in full
**2**	**rayyan-250332020**	Venturini et al	A 3D virtual environment for empirical research on social pain: Enhancing fidelity and anthropomorphism in the study of feelings of ostracism inclusion and overinclusion	Not about a vulnerable population (minority)
**3**	**rayyan-250332082**	Yan et al	A brief online mindfulness intervention: study protocol for Indonesian undergraduate students, a randomized controlled trial	Not about a vulnerable population (minority)
**4**	**rayyan-250332652**	Baruah et al	A Comparison of Online and Offline Support Modalities as Predictors of Stress Among Traditional and Non-Traditional College Students	Duplication
**5**	**rayyan-250333217**	Hinduan et al	A Counseling Application as an Alternative Tool in Increasing Coping Self-Efficacy Among University Students With Academic Distress During Coronavirus Disease 2019 Pandemic in Indonesia: A Study Protocol for a Randomized Controlled Non-Inferiority Trial	Not about a vulnerable population (minority)
**6**	**rayyan-250333120**	Suasthi	A holistic approach to student well-being in Hindu education: The effects of spiritual teaching practices, community involvement, and classroom environment	No technological intervention
**7**	**rayyan-250333135**	Brogly et al	A mobile app to identify lifestyle indicators related to undergraduate mental health (smart healthy campus): Observational app-based ecological momentary assessment	Not about a vulnerable population (minority)
**8**	**rayyan-250332002**	Fuller et al	A Mobile App-Based Gratitude Intervention's Effect on Mental Well-Being in University Students: Randomized Controlled Trial	Not about a vulnerable population (minority)
**9**	**rayyan-250332017**	Bendtsen et al	A Mobile Health Intervention for Mental Health Promotion Among University Students: Randomized Controlled Trial	Not about a vulnerable population (minority)
**10**	**rayyan-250332985**	AlZu'bi et al	A Novel Deep Learning Technique for Detecting Emotional Impact in Online Education	Not about a vulnerable population (minority)

**11**	**rayyan-250332167**	Bangalan and Agnes	A pilot study of the AKBAY mobile app for the mental health of university students	Article not available in full
**12**	**rayyan-250332994**	Kertechian and Ismail	A Positive View of Excessive Smartphone Utilization and Its Relationship With Other Academic-Related Variables Within the Online Course Setting	Article not available in full
**13**	**rayyan-250333046**	Larsson et al	A randomised controlled trial of brief web-based acceptance and commitment Therapy on the general mental health, depression, anxiety and stress of college Students	Not about a vulnerable population (minority)
**14**	**rayyan-250332624**	Zimmermann and Papa	A randomized controlled trial of a brief internet intervention to prevent anxiety and depression among college students	Not about a vulnerable population (minority) Duplication
**15**	**rayyan-250331746**	Nguyen-Feng	A randomized controlled trial of a mobile ecological momentary stress management intervention for students with and without a history of emotional abuse	Not about a vulnerable population (minority)
**16**	**rayyan-250332491**	Bedesem and Barber	A Teacherâ€™s Guide to Technology-Based Self-Monitoring Strategies for Student Behavior	Not about university students
**17**	**rayyan-250332707**	López-Padrón et al	Academic use of smartphones in postgraduate education: student perception in Ecuador	Article not available in full
**18**	**rayyan-250333178**	Eisenstadt et al	Acceptability, engagement, and exploratory outcomes of an emotional well-being app: Mixed methods preliminary evaluation and descriptive analysis	Not about university students
**19**	**rayyan-250331614**	Lira et al	Adaptation of a mobile app for early anxiety and depression intervention in university students in Chile: Participatory study.	Not about a vulnerable population (minority)
**20**	**rayyan-250332290**	Saccardi and Masthoff	Adapting emotional support in teams: productivity, emotional stability, and conscientiousness	Not about a vulnerable population (minority)
**21**	**rayyan-250333232**	Global	Addressing the Covid-19 Burden on Medical Education and Training: The Role of Telemedicine and Tele-Education During and Beyond the Pandemic	Narrative review
**22**	**rayyan-250333359**	Olasupo and Idemudia	Adjustment, psychological well-being and mental health of first year students in a South African university	Although it is an African study, it does not address the vulnerability of social minorities
**23**	**rayyan-250332771**	Villa-Enciso et al	Agent-Based Model to Analyze the Role of the University in Reducing Social Exclusion	Not about a vulnerable population (minority)

**24**	**rayyan-250333323**	Mahoney	Aging anxieties and disturbed eating in female students: Itâ€™s not all about aging appearance concern	Not about a vulnerable population (minority)
**25**	**rayyan-250333011**	Patel et al	An exploration in to how young-people from ethnic-minority backgrounds interact with online counselling	Article not available in full
**26**	**rayyan-250332431**	Candelario Navarrete et al	Analysis of poverty as a factor in the dropout rate of university students in Mexico City from 2000 to 2022	No technological intervention
**27**	**rayyan-250332813**	Cortés-Rodríguez et al	Analysis of Psychological Well-Being from a Compositional Data Analysis Perspective: A New Approach	Not about a vulnerable population (minority)
**28**	**rayyan-250333454**	Omheni et al	Annotation-based learner's personality modeling in distance learning context	Not about a vulnerable population (minority)
**29**	**rayyan-250332803**	Salva et al	Anxiety and Digital Phenotypes as Diversity Markers for Selected Filipino University Students	Not about a vulnerable population (minority)
**30**	**rayyan-250333123**	Herbert et al	Are You Willing to Self-Disclose for Science? Effects of Privacy Awareness and Trust in Privacy on Self-Disclosure of Personal and Health Data in Online Scientific Studiesâ€”An Experimental Study	Does not contain a minority population
**31**	**rayyan-250332371**	Zhang et al	Association between social media use and studentsâ€™ academic performance through family bonding and collective learning: The moderating role of mental well-being	Not about a vulnerable population (minority)
**32**	**rayyan-250332881**	Feng et al	Associations of Death Anxiety, Inclusion of Smartphone in the Self, and Affiliation Motivation with Smartphone use and Addiction: A Multiple Mediation Study	Not about a vulnerable population (minority)
**33**	**rayyan-250332992**	Nogueira-Leite and Cruz-Correia	Attitudes of Physicians and Individuals Toward Digital Mental Health Tools: Protocol for a Web-Based Survey Research Project	Not about a vulnerable population (minority)
**34**	**rayyan-250333233**	Světlák et al	Being Mindful at University: A Pilot Evaluation of the Feasibility of an Online Mindfulness-Based Mental Health Support Program for Students	Contains a minority population
**35**	**rayyan-250332966**	Cormier et al	College Students eMental Health Literacy and Risk of Diagnosis with Mental Health Disorders	Not about a vulnerable population (minority)
**36**	**rayyan-250332664**	Pandya	College students with high abilities in liberal arts disciplines: Examining the effect of spirituality in bolstering self-regulated learning, affect balance, peer relationships, and well-being	Not about a vulnerable population (minority)

**37**	**rayyan-250332610**	Adedokun and Popoola	Computer Self-efficacy, Computer Literacy Skills, Cognitive Skills and Use of Electronic Resources by Social Science Doctoral Students in Nigerian Federal Universities	Not about a vulnerable population (minority)
**38**	**rayyan-250333452**	Maindal et al	Cultural adaptation and validation of the Health Literacy Questionnaire (HLQ): robust nine-dimension Danish language confirmatory factor model	Not about a vulnerable population (minority)
**39**	**rayyan-250333446**	Apolinário-Hagen et al	Current views and perspectives on e-mental health: An exploratory survey study for understanding public attitudes toward internet-based psychotherapy in Germany	Not about a vulnerable population (minority)
**40**	**rayyan-250332792**	Khalijian et al	Customization and use of digital storytelling in providing online career counseling services to students with physical-motor disabilities: A mixed study	Not about a vulnerable population (minority)
**41**	**rayyan-250332484**	Pang	Determining the influence of depressive mood and self-disclosure on problematic mobile app use and declined educational attainment: Insight from stressor-strain-outcome perspective	Not about a vulnerable population (minority)
**42**	**rayyan-250333184**	Ryabchikova et al	Development of students' verbal creativity using flash cards in foreign language classes	Not about a vulnerable population (minority)
**43**	**rayyan-250333325**	Fullagar et al	Digital ecologies of youth mental health: Apps, therapeutic publics and Pedagogy as affective arrangements	Not about a vulnerable population (minority) and does not focus on university
**44**	**rayyan-250332579**	Kang and Hong	Digital Interventions for Reducing Loneliness and Depression in Korean College Students: Mixed Methods Evaluation	Not about a vulnerable population (minority)
**45**	**rayyan-250332978**	Niu et al	Digital learning of English as a foreign language among university students: How are approaches to learning linked to digital competence and technostress?	Not about a vulnerable population (minority)
**46**	**rayyan-250332047**	Amoako and Anane	Digital teaching competence and resilience across tutors of different age and gender in Ghana	Not university students
**47**	**rayyan-250333057**	Bai et al	Effect of Perceived Fear, Quality, and Self-Determination on Learnersâ€™ Retention Intention on MOOCs	Not about a vulnerable population (minority)
**48**	**rayyan-250333176**	Bunyakul et al	Effects of a mobile game on students' learning achievements and motivations in a clinical chemistry course: learning style differences	Article not available in full

**49**	**rayyan-250332502**	Selim et al	Effects of Large Language Modelâ€“Based Offerings on the Well-Being of Students: Qualitative Study	Not about a vulnerable population (minority)
**50**	**rayyan-250332076**	Bowers et al	Evaluating the naturalistic implementation of a peer-coaching service to augment online acceptance and commitment therapy for college mental health	Not about a vulnerable population (minority)
**51**	**rayyan-250332265**	Coffield and Kausar	Evaluating User Engagement With a Real-Time, Text-Based Digital Mental Health Support App: Cross-Sectional, Retrospective Study	Not about a vulnerable population (minority)
**52**	**rayyan-250333421**	Zhao and Pardo	Evolving the design of a mobile application to support transition to tertiary education	Not about a vulnerable population (minority)
**53**	**rayyan-250332607**	Farhi	Examining the factors fostering metaverse experience browser acceptance under unified theory of acceptance and use of technology (UTAUT)	Not about a vulnerable population (minority)
**54**	**rayyan-250333355**	Jiang and Li	Factors affecting smartphone dependency among the young in China	Not an intervention for welfare
**55**	**rayyan-250332100**	Shahzad et al	Generative Artificial Intelligence (ChatGPT-4) and Social Media Impact on Academic Performance and Psychological Well-Being in China's Higher Education	Not about a vulnerable population (minority)
**56**	**rayyan-250332128**	Cengiz and Peker	Generative artificial intelligence acceptance and artificial intelligence anxiety among university students: the sequential mediating role of attitudes toward artificial intelligence and literacy	Not about a vulnerable population (minority)
**57**	**rayyan-250333389**	Odacl and Çelik	Group Counselling on College Students' Internet Dependency and Life Satisfaction	Not about a vulnerable population (minority)
**58**	**rayyan-250332425**	Rahman and Saleem	HABIT AND HEDONISM: SMARTPHONE DEPENDENCY BEYOND UTILITY	Article not available in full
**59**	**rayyan-250332653**	Haddow and Brodie	Harnessing innovation approaches to support community and belonging in Higher Education	No technological intervention
**60**	**rayyan-250332227**	Kuhail et al	Human vs. AI counseling: College students' perspectives	Not about a vulnerable population (minority)
**61**	**rayyan-250333092**	Harkin et al	I Want to See People's Reactions to the Selfiesâ€: A Lefebvrian Analysis of the Impact of Social Networking Sites on Physical, Mental, and Emotional Functioning	Not about a vulnerable population (minority)
**62**	**rayyan-250332376**	Gutiérrez-Huancayo	Impact of a YouTube Channel on ESL Students' Oral Skills in a Peruvian Higher Institute	Not about a vulnerable population (minority)

**63**	**rayyan-250332003**	Six et al	Impact of Conversational and Animation Features of a Mental Health App Virtual Agent on Depressive Symptoms and User Experience Among College Students: Randomized Controlled Trial	Not about a vulnerable population (minority)
**64**	**rayyan-250332397**	Li et al	Impact of Mobile Phone Use on College Studentsâ€™ Self-perceived Health During the Epidemic in China: The Mediating Role of Sleep Quality	Article not available in full
**65**	**rayyan-250332709**	Ochoa-Cueva et al	Implementing the CLIL Approach through Online Games in EFL Education	Not about a vulnerable population (minority)
**66**	**rayyan-250332537**	Gill et al	Implementing Universal Design through augmented-reality game-based learning	Not about mental health or well-being
**67**	**rayyan-250332840**	Girio-Herrera et al	Improving Black student outcomes: The multifaceted role of psychology professors in higher education	No technological intervention
**68**	**rayyan-250331613**	Robledo Yamamoto	Incorporating community in the design of mental health technologies.	Not university students
**69**	**rayyan-250333382**	Ervasti et al	Influence of personality and differences in stress processing among Finnish students on interest to use a mobile stress management app: Survey study	Not about a vulnerable population (minority)
**70**	**rayyan-250333043**	Li	Internet Entrepreneurship Psychology for College Students and Internet Financial Crimes Prevention	Not about a vulnerable population (minority)
**71**	**rayyan-250332958**	Jafarlou et al	Investigar o uso, as atitudes e a intenção de universitários sul-africanos em utilizar soluções digitais para saúde mental, e fatores associados à intenção de uso.	Duplication
**72**	**rayyan-250332639**	Ahmed et al	Investigating Rhythmicity in App Usage to Predict Depressive Symptoms: Protocol for Personalized Framework Development and Validation Through a Countrywide Study	Not about a vulnerable population (minority)
**73**	**rayyan-250332230**	Li and Lyu	Leveraging Mobile Technology for Enhanced Mental Health Levels in University Students	Not about a vulnerable population (minority)
**74**	**rayyan-250332051**	Raimann et al	Male Students in Female-Dominated Study Programs: Perceived Diversity Climate in Gender-Mixed Computer-Supported Collaborative Learning	Not about a vulnerable population (minority)
**75**	**rayyan-250333071**	Burmicky et al	Mattering and Belonging: An HBCU Case Study Exploration of Campus Involvement during the Pandemic	Article not available in full

**76**	**rayyan-250333418**	Zhao and Wang	Mediated hook-up: Gratifications and psychological attributes as predictors of Chinese college studentsâ€™ hook-up behaviors	Not about a vulnerable population (minority)
**77**	**rayyan-250332202**	Ying	Mental Health Assessment Model for College Students Using Circular q-Rung Orthopair Fuzzy Muirhead Means and MULTIMOORA Method	Not about a vulnerable population (minority)
**78**	**rayyan-250332569**	Tangco-Siason and Siason	Mental Health Competence of Faculty in a State University	Not about a vulnerable population (minority)
**79**	**rayyan-250332679**	Gao	Mindfulness and foreign language learners self-perceived proficiency: the mediating roles of anxiety and burnout	Not about a vulnerable population (minority)
**80**	**rayyan-250332933**	Aboelmaged et al	Mobile apps use for wellness and fitness and university students' subjective wellbeing	Duplication
**81**	**rayyan-250332519**	Nechyporenko et al	Mobile technologies as a tool to support inclusive learning in higher education institutions	Not about a vulnerable population (minority)
**82**	**rayyan-250333257**	Niyigena et al	Modeling the measurements of the determinants of ICT fluency and evolution of digital divide among students in developing countries-East Africa case study	Not about mental health or well-being
**83**	**rayyan-250332048**	Tovmasyan et al	Motivating Transparent Communications About Bias in Healthcare Technology Development	Not about a vulnerable population (minority)
**84**	**rayyan-250333076**	Sifat et al	Motivations Toward Using Digital Health and Exploring the Possibility of Using Digital Health for Mental Health in Bangladesh University Students: Cross-sectional Questionnaire Study	Not about a vulnerable population (minority)
**85**	**rayyan-250332934**	Deniz and Özek	Online Learning Experiences of Graduate Students in TÃ¼rkiye: Could This Be the Footsteps of a Reform?	Not about a vulnerable population (minority)
**86**	**rayyan-250332997**	Chambi et al	Perceived Motivational Effects of Mobile Learning Technique to Higher Education Students: An Exploratory Study	Not about a vulnerable population (minority)
**87**	**rayyan-250332098**	Brockmeier et al	Planning a digital detox: Findings from a randomized controlled trial to reduce smartphone usage time	Not about a vulnerable population (minority)
**88**	**rayyan-250333165**	Valenzuela-García et al	Positive school environment, sustainable behaviour and well-being among higher education students (Ambiente escolar positivo, conducta sostenible ybienestar en estudiantes de educaciÃ³n superior)	Article not available in full
**89**	**rayyan-250333292**	Aborujiah et al	Post acceptance model for online teleconsultation services: An empirical study in Malaysia	Not about a vulnerable population (minority)

**90**	**rayyan-250332324**	Yang et al	Predictors of health preventive behavior among university students in the post-COVID-19 era in Wuhan via TikTok journeying	Not about a vulnerable population (minority)
**91**	**rayyan-250332770**	Lucas-Thompson et al	Preliminary Evaluation of Learning to BREATHE PLUS for University Students: Does a Multi-Modal Adaptive Supplement Strengthen Effects of a Mindfulness-Based Intervention?	Article not available in full
**92**	**rayyan-250333089**	Liu et al	Problems and countermeasures associated with intercultural adaptation in international education according to the communication action theory model	No technological intervention
**93**	**rayyan-250333068**	Sorkhou et al	Psychological Distress in Treatment-Seeking University Students: An Intersectional Examination of Asian Identity and Gender Identity	Article not available in full
**94**	**rayyan-250333048**	Yan et al	Psychometric Properties of the Chinese Revision of the Pitt Wellness Scale for People in the University Environment	No technological intervention
**95**	**rayyan-250332106**	Elvira-Zorzo et al	Psychosocial Differences Between Female and Male Students in Learning Patterns and Mental Health-Related Indicators in STEM vs. Non-STEM Fields	Not about a vulnerable population (minority)
**96**	**rayyan-250332742**	Saleh and Al-Ali	Psycho-Technological Compatibility as One of the Guidelines for Students of Computer Science	Not about a vulnerable population (minority)
**97**	**rayyan-250333453**	Omheni et al	Recognition of learner's personality traits through digital annotations in distance learning	Article not available in full
**98**	**rayyan-250333069**	Meng	Role of politically motivated internet addiction and ideological passion in linking college student's mental health education and wellbeing	Not about a vulnerable population (minority)
**99**	**rayyan-250332382**	Kleinpeter et al	Satisfaction and Therapeutic Alliance with Mental Health Providers in Teletherapy as Opposed to Face-to-Face Counseling for College Students: Diversity, Equity, Inclusion, and Accessibility	Article not available in full
**100**	**rayyan-250333270**	Parker et al	School Mental Health Traineesâ€™ Perceptions of a Virtual Community-Based Partnership to Support Black Youth	Article not available in full
**101**	**rayyan-250332886**	Zhu et al	Self-perception evolution among university student TikTok users: evidence from China	Not about a vulnerable population (minority)

**102**	**rayyan-250333158**	Martino et al	Sinapsi Academic Self-Management Training Group to Promote Well-Being with University Students	Not about a vulnerable population (minority)
**103**	**rayyan-250333284**	Sa et al	Sleep Duration and Weight Gain among Students at a Historically Black University	Article not available in full
**104**	**rayyan-250332941**	Sharma et al	Smartphone-induced Behaviour: Utilisation, Benefits, Nomophobic Behaviour and Perceived Risks	Does not contain a minority population
**105**	**rayyan-250332689**	Huang et al	Specific Internet Disorders in University Students in Taiwan and Hong Kong: Psychometric Properties with Invariance Testing for the Traditional Chinese Version of the Assessment of Criteria for Specific Internet-Use Disorders (ACSID-11)	Not about a vulnerable population (minority)
**106**	**rayyan-250332162**	Aldowah et al	Students' online engagement in higher education: a DEMATEL study	Not about a vulnerable population (minority)
**107**	**rayyan-250332999**	Sanders	Support and Aid to Families Electronically (SAFE): Addressing Intersecting Academic and Community Needs	Not about a vulnerable population (minority)
**108**	**rayyan-250332949**	Esposito et al	Supporting University Students During the Pandemic: A Study on The Efficacy of a Mentalizing Online Group Counselling	Not about a vulnerable population (minority)
**109**	**rayyan-250333425**	Longo et al	Technology Use, Exposure to Natural Hazards, and Being Digitally Invisible: Implications for Policy Analytics	Not about the mental health of university students, but rather of homeless people
**110**	**rayyan-250332745**	Suherman et al	Technology-Based Intervention for Building Healthy Campus: Health Promoting Lifestyle in the Universities	Not about a vulnerable population (minority)
**111**	**rayyan-250332242**	Zhang and Mo	Technology-Facilitated Sexual Violence Among Chinese University Students: Victimization, Perpetration, and Association with Mental Health	Not about a vulnerable population (minority)
**112**	**rayyan-250333110**	Ajlouni and Rawadieh	Technophobia and Technophilia among Undergraduates: Cross-national Research in Jordan, Qatar, and Egypt	Not about a vulnerable population (minority)
**113**	**rayyan-250332490**	Rettinger et al	Telehealth Education in Allied Health Care and Nursing: Web-Based Cross-Sectional Survey of Studentâ€™s Perceived Knowledge, Skills, Attitudes, and Experience	Not about a vulnerable population (minority)
**114**	**rayyan-250333353**	Payton et al	Text mining mental health reports for issues impacting today's college students: Qualitative study	Literature review
**115**	**rayyan-250333450**	Lekka et al	The effect of counselling-based training on online peer support	Not about a vulnerable population (minority)

**116**	**rayyan-250332299**	Tinella et al	The effects of university psychological counseling on studentsâ€™ mental health in the COVID- 19 pandemic era	Not about a vulnerable population (minority)
**117**	**rayyan-250332847**	Darban	The future of virtual team learning: navigating the intersection of AI and education	Article not available in full
**118**	**rayyan-250332766**	Fangling et al	THE IMPACT OF PERCEPTION AND IMMERSION ON POSITIVE EMOTION TOWARDS 360 PANORAMIC ANIMATION AMONG UNIVERSITY STUDENTS-A STRUCTURAL EQUATION MODELING	Not about a vulnerable population (minority)
**119**	**rayyan-250332740**	He et al	The influence of educational and emotional support on e-learning acceptance: An integration of social support theory and TAM	Not about a vulnerable population (minority)
**120**	**rayyan-250333169**	Zhang et al	The Moderating Role of Grit in the Relationship Between Perfectionism and Depression Among Chinese College Students	Not about a vulnerable population (minority)
**121**	**rayyan-250333362**	Yu et al	The potential of Second Life for university counseling: a comparative approach examining media features and counseling problems	Not about a vulnerable population (minority)
**122**	**rayyan-250333338**	Amir Hamzah et al	The Prevalence and Associated Factors of Depression, Anxiety and Stress of First Year Undergraduate Students in a Public Higher Learning Institution in Malaysia	Not about a vulnerable population (minority)
**123**	**rayyan-250333096**	Oweis	The role of social media in promoting citizenship values of international students during the COVID-19 global health crisis	Article not available in full
**124**	**rayyan-250332663**	Cogan et al	The taboo of mental health problems, stigma and fear of disclosure among Asian international students: implications for help-seeking, guidance and support	No technological intervention
**125**	**rayyan-250333175**	Rodriguez et al	The use of task shifting to improve treatment engagement in an internet-based mindfulness intervention among Chinese university students: Randomized controlled trial	Not about a vulnerable population (minority)
**126**	**rayyan-250332347**	Ruzek et al	Training the Psychologist of the Future in the Use of Digital Mental Health Technologies	Not about a vulnerable population (minority)
**127**	**rayyan-250332010**	Primasari et al	Trauma web-based psychoeducational programme: systematic cultural adaptation and protocol for a feasibility-acceptability study	Not about a vulnerable population (minority)

**128**	**rayyan-250332336**	Şahin and Yıldız	Understanding mobile learning acceptance among university students with special needs: An exploration through the lens of self-determination theory	Not about a vulnerable population (minority)
**129**	**rayyan-250331684**	Bernardes	Universidade, saÃºde mental e direitos humanos: uma anÃ¡lise institucional a partir das vivÃªncias dos estudantes da Universidade Federal do Tocantins	No technological intervention
**130**	**rayyan-250333053**	Şahin et al	University students with special needs: Investigating factors influencing e-learning adoption	Not about a vulnerable population (minority)
**131**	**rayyan-250332055**	Shah et al	Unraveling the post-adoptive hybrid learning experience: perspectives from information systems	Article not available in full
**132**	**rayyan-250332252**	Wittmar et al	User Experience With a Personalized mHealth Service for Physical Activity Promotion in University Students: Mixed Methods Study	Not about a vulnerable population (minority)
**133**	**rayyan-250332451**	Stanger and Lucas	Using Indirect Service-Learning to Promote Evidence-Based Digital Mental Health Tools on College Campuses	Not about a vulnerable population (minority)
**134**	**rayyan-250333460**	Yang et al	Using mobile phones in college classroom settings: Effects of presentation mode and interest on concentration and achievement	Not about a vulnerable population (minority)
**135**	**rayyan-250332965**	Woo	Utilising K-culture for Korean college students writing identity essays: online class cases	Article not available in full
**136**	**rayyan-250332087**	Yensathit et al	Virtual reality as a tool to alleviate library anxiety and promote service knowledge acquisition among visually impaired students	Article not available in full
**137**	**rayyan-250332229**	Ho et al	Young People's Preferences for Web-Based Mental Health Interventions for Managing Anxiety and Depression: A Discrete Choice Experiment	Not about a vulnerable population (minority)

Supplementary Table 4 - Full-Text Studies Excluded and Reasons

6 Complete search strategy

6.1 PubMed/MEDLINE

("dhts" OR "dht" OR "digital mental health" OR "semantic interoperability" OR "Interoperability" OR "Health Information Interoperability" OR "digital health" OR "telemedicine" OR "telehealth" OR "mHealth" OR "e-health" OR "digital intervention*" OR "mobile application*" OR "web-based intervention*" OR "online counseling" OR "digital education intervention*" OR "digital psychoeducation" OR "digital platform*" OR "technology-based intervention*") AND ("mental health" OR "well-being" OR "psychological well-being" OR "anxiety" OR "depression" OR "psychological distress" OR "mental wellness" OR "emotional support") AND ("affirmative action" OR "quota students" OR "racial equity") AND ("racism" OR "ethnic discrimination" OR "structural racism" OR "institutional racism" OR "racial discrimination" OR "health status disparities" OR "black population health" OR "racial bias" OR "racial injustice" OR "black students") AND ("university students" OR "higher education" OR "undergraduate students" OR "academic health")

6.2 Embase

('digital mental health intervention*' OR 'digital mental health'/exp OR 'digital mental health' OR 'online counseling'/exp OR 'online counseling' OR 'mhealth'/exp OR 'mhealth' OR 'mobile application'/exp OR 'mobile application' OR 'web-based intervention'/exp OR 'web-based intervention' OR 'digital psychoeducation' OR 'e-health'/exp OR 'e-health' OR 'telemedicine'/exp OR 'telemedicine') AND ('mental health'/exp OR 'mental health' OR 'psychological distress'/exp OR 'psychological distress' OR 'well-being'/exp OR 'well-being' OR 'emotional support'/exp OR 'emotional support' OR 'depression'/exp OR 'depression' OR 'anxiety'/exp OR 'anxiety' OR 'psychological well-being'/exp OR 'psychological well-being' OR 'emotional health'/exp OR 'emotional health') AND ('affirmative action'/exp OR 'affirmative action' OR 'quota students' OR 'college admissions' OR 'inclusion policy' OR 'racism'/exp OR 'racism' OR 'structural racism'/exp OR 'structural racism' OR 'racial discrimination'/exp OR 'racial discrimination' OR 'racial equity'/exp OR 'racial equity' OR 'ethnic discrimination'/exp OR 'ethnic discrimination' OR 'institutional racism'/exp OR 'institutional racism' OR 'racial bias'/exp OR 'racial bias' OR 'racial injustice' OR 'health status disparities'/exp OR 'health status disparities' OR 'inequity' OR 'inequality'/exp OR 'inequality' OR 'diversity'/exp OR 'diversity' OR 'minority students' OR 'students of color' OR 'african american students' OR 'black students' OR 'bipoc students' OR

marginalized OR underrepresented OR 'inclusion'/exp OR inclusion OR 'anti blackness') AND ('university students' OR 'college students'/exp OR 'college students' OR 'undergraduate students' OR 'higher education'/exp OR 'higher education' OR 'graduate students' OR 'student retention'/exp OR 'student retention' OR 'academic achievement'/exp OR 'academic achievement') AND 'article'/it AND ([english]/lim OR [portuguese]/lim OR [spanish]/lim) AND [2015-2025]/py

6.3 Scopus

("digital mental health intervention*" OR "digital mental health" OR "online counseling" OR "mHealth" OR "mobile application" OR "web-based intervention" OR "digital psychoeducation" OR "e-health" OR "telemedicine") AND ("mental health" OR "psychological distress" OR "well-being" OR "emotional support" OR "depression" OR "anxiety" OR "psychological well-being" OR "emotional health") AND ("affirmative action" OR "quota students" OR "college admissions" OR "inclusion policy" OR "racism" OR "structural racism" OR "racial discrimination" OR "racial equity" OR "ethnic discrimination" OR "institutional racism" OR "racial bias" OR "racial injustice" OR "health status disparities" OR "inequity" OR "inequality" OR "diversity" OR "minority students" OR "students of color" OR "African American students" OR "black students" OR "BIPOC students" OR marginalized OR underrepresented OR inclusion OR anti-blackness) AND ("university students" OR "college students" OR "undergraduate students" OR "higher education" OR "graduate students" OR "student retention" OR "academic achievement") AND PUBYEAR > 2014 AND PUBYEAR < 2026 AND (LIMIT-TO (DOCTYPE , "ar")) AND (LIMIT-TO (LANGUAGE , "English") OR LIMIT-TO (LANGUAGE , "Spanish") OR LIMIT-TO (LANGUAGE , "Portuguese")) AND (EXCLUDE (EXACTKEYWORD , "Adolescent") OR EXCLUDE (EXACTKEYWORD , "Child") OR EXCLUDE (EXACTKEYWORD , "Systematic Review") OR EXCLUDE (EXACTKEYWORD , "SARS-CoV-2") OR EXCLUDE (EXACTKEYWORD , "Exercise") OR EXCLUDE (EXACTKEYWORD , "Clinical Article") OR EXCLUDE (EXACTKEYWORD , "Human") OR EXCLUDE (EXACTKEYWORD , "Humans") OR EXCLUDE (EXACTKEYWORD , "Article") OR EXCLUDE (EXACTKEYWORD , "Female") OR EXCLUDE (EXACTKEYWORD , "Adult") OR EXCLUDE (EXACTKEYWORD , "Male") OR EXCLUDE (EXACTKEYWORD , "Middle Aged") OR EXCLUDE (EXACTKEYWORD , "Aged") OR EXCLUDE (EXACTKEYWORD , "Obesity") OR EXCLUDE (EXACTKEYWORD , "Demography") OR EXCLUDE (EXACTKEYWORD , "Older Adults") OR EXCLUDE (EXACTKEYWORD , "Very Elderly") OR EXCLUDE (EXACTKEYWORD , "Aging") OR EXCLUDE (EXACTKEYWORD , "Aged, 80 And Over") OR EXCLUDE (EXACTKEYWORD , "Demographics") OR EXCLUDE (EXACTKEYWORD , "Physical Activity") OR EXCLUDE (EXACTKEYWORD , "COVID-19") OR EXCLUDE (EXACTKEYWORD , "Behavioral Research") OR EXCLUDE (EXACTKEYWORD , "Pandemic") OR EXCLUDE (EXACTKEYWORD , "COVID-19 Pandemic") OR EXCLUDE (EXACTKEYWORD , "Adolescents") OR EXCLUDE (EXACTKEYWORD , "Surveys") OR EXCLUDE (EXACTKEYWORD , "Gender") OR EXCLUDE (EXACTKEYWORD , "Systematic Literature Review") OR EXCLUDE (EXACTKEYWORD , "Covid-19") OR EXCLUDE (EXACTKEYWORD , "'current") OR EXCLUDE (EXACTKEYWORD

, "Alcohol") OR EXCLUDE (EXACTKEYWORD , "Artificial Intelligence") OR EXCLUDE (EXACTKEYWORD , "Gamification") OR EXCLUDE (EXACTKEYWORD , "Machine Learning") OR EXCLUDE (EXACTKEYWORD , "Sustainability") OR EXCLUDE (EXACTKEYWORD , "Perceived Usefulness") OR EXCLUDE (EXACTKEYWORD , "Content Analysis") OR EXCLUDE (EXACTKEYWORD , "Artificial Neural Network") OR EXCLUDE (EXACTKEYWORD , "Communication") OR EXCLUDE (EXACTKEYWORD , "Big Data") OR EXCLUDE (EXACTKEYWORD , "AI") OR EXCLUDE (EXACTKEYWORD , "Structural Equation Modeling") OR EXCLUDE (EXACTKEYWORD , "Metaverse") OR EXCLUDE (EXACTKEYWORD , "Trust") OR EXCLUDE (EXACTKEYWORD , "Sustainable Development") OR EXCLUDE (EXACTKEYWORD , "Youth") OR EXCLUDE (EXACTKEYWORD , "Quality Of Life") OR EXCLUDE (EXACTKEYWORD , "Perception") OR EXCLUDE (EXACTKEYWORD , "Health") OR EXCLUDE (EXACTKEYWORD , "Education") OR EXCLUDE (EXACTKEYWORD , "Students") OR EXCLUDE (EXACTKEYWORD , "Bisexual") OR EXCLUDE (EXACTKEYWORD , "Gay") OR EXCLUDE (EXACTKEYWORD , "Adolescence") OR EXCLUDE (EXACTKEYWORD , "Women") OR EXCLUDE (EXACTKEYWORD , "Meta-analysis") OR EXCLUDE (EXACTKEYWORD , "Attitudes"))

6.4 Web of Science

("digital mental health intervention*" OR "digital mental health" OR "online counseling" OR "mHealth" OR "mobile application" OR "web-based intervention" OR "digital psychoeducation" OR "e-health" OR "telemedicine")
AND
("mental health" OR "psychological distress" OR "well-being" OR "emotional support" OR "depression" OR "anxiety" OR "psychological well-being" OR "emotional health")
AND
("affirmative action" OR "quota students" OR "college admissions" OR "inclusion policy" OR "racism" OR "structural racism" OR "racial discrimination" OR "racial equity" OR "ethnic discrimination" OR "institutional racism" OR "racial bias" OR "racial injustice" OR "health status disparities" OR "inequity" OR "inequality" OR "diversity" OR "minority students" OR "students of color" OR "African American students" OR "black students" OR "BIPOC students" OR marginalized OR underrepresented OR inclusion OR anti-blackness)
AND
("university students" OR "college students" OR "undergraduate students" OR "higher education" OR "graduate

6.5 PsycINFO

("digital mental health intervention*" OR "digital mental health" OR "online counseling" OR "mHealth" OR "mobile application" OR "web-based intervention" OR "digital psychoeducation" OR "e-health" OR "telemedicine")
AND
("mental health" OR "psychological distress" OR "well-being" OR "emotional support" OR

"depression" OR "anxiety" OR "psychological well-being" OR "emotional health")
AND
("affirmative action" OR "quota students" OR "college admissions" OR "inclusion policy" OR "racism" OR "structural racism" OR "racial discrimination" OR "racial equity" OR "ethnic discrimination" OR "institutional racism" OR "racial bias" OR "racial injustice" OR "health status disparities" OR "inequity" OR "inequality" OR "diversity" OR "minority students" OR "students of color" OR "African American students" OR "black students" OR "BIPOC students" OR marginalized OR underrepresented OR inclusion OR anti-blackness)
AND
("university students" OR "college students" OR "undergraduate students" OR "higher education" OR "graduate

6.6 Google Scholar

In the "all words" field of the Google Scholar search, we do the following:

In Portuguese:
dht + e-saúde e-educação digital intervenção bem-estar tecnologias saúde mental cotas racismo ações afirmativas universidade estudantes negros

In English:
dht + digital intervention technologies mental health racism affirmative action university black students
eHealth m-health

In Spanish:
dht + intervención digital, tecnologías de bienestar, salud mental, racismo, acción afirmativa, estudiantes negros universitarios

dht in Portuguese:
-telessaúde
-telemedicina
-aplicativos móveis
-interoperabilidade
-terapia assistida por computador
-plataformas virtuais

dht in English:
-telehealth
-telemedicine
-interoperability
-platforms
-virtual care
-health IT

dht in Spanish:
-telesalud
-telemedicine
-interoperabilidad

-atención virtual
-salud en línea
-aplicaciones móviles

7 Descriptors, Keywords, and "Single" Boolean Logic

Block	Descriptors in square brackets and keywords.
Digital EduHealth	["digital health" OR "e-health" OR "telemedicine" OR "mHealth" OR "Health Information Interoperability" OR "Technology-based Intervention" OR "Online Counseling"] OR dhts OR dht OR digital OR DMHI OR "digital intervention*" OR "digital mental health" OR "mobile application*" OR "web-based intervention*" OR "digital education intervention*" OR "digital psychoeducation" OR "digital platform*" OR "mobile health" OR technology OR "Digital mental health interventions" OR platform* OR apps OR "Ecological Momentary Intervention" OR "Ecological Momentary Assessment" OR "online support" OR "self-help" OR codesign
Mental Health/Well-being	["mental health" OR "anxiety" OR "depression" OR "psychological distress" OR "well-being" OR "emotional support" OR "psychological well-being" OR "mental disorders"] OR mental OR distress OR "mental wellness" OR disorder OR illness OR emotional OR empathy OR stigma OR struggle OR support OR "mental well-being" OR "mental illness" OR "emotional health" OR shame
Affirmative Action/Racism	["affirmative action" OR "racial equity" OR "racism" OR "institutional racism" OR "structural racism" OR "racial discrimination" OR "ethnic discrimination" OR "minority groups"] OR "black students" OR "students of color" OR ethnicity OR ethnicities OR ethnic OR Étnico OR Étnica OR Etnicidade OR "quota students" OR cotistas OR racial OR race OR "racial bias" OR "racial injustice" OR inequity OR inequality OR "health status disparities" OR "anti-blackness OR underrepresented OR diversity OR marginalized OR inclusion OR "black support"
University Students	["university students" OR "higher education" OR "undergraduate students" OR "college students" OR "academic achievement" OR "student retention" OR "Health Services in Universities" OR " OR "Student Dropouts"] OR university OR academic OR college OR undergraduate OR "academic health" OR "black doctoral students" OR "graduate students" OR "college students of color"

**Boolean Logic**	**(<br>"digital health" OR "e-health" OR "telemedicine" OR "mHealth" OR "Health Information Interoperability" OR "Technology-based Intervention" OR "Online Counseling" OR dhts OR dht OR digital OR DMHI OR "digital intervention*" OR "digital mental health" OR "mobile application*" OR "web-based intervention*" OR "digital education intervention*" OR "digital psychoeducation" OR "digital platform*" OR "mobile health" OR technology OR "Digital mental health interventions" OR platform* OR apps OR "Ecological Momentary Intervention" OR "Ecological Momentary Assessment" OR "online support" OR "self-help" OR codesign<br>)<br>AND<br>(<br>"mental health" OR "anxiety" OR "depression" OR "psychological distress" OR "well-being" OR "emotional support" OR "psychological well-being" OR "mental disorders" OR mental OR distress OR "mental wellness" OR disorder OR illness OR emotional OR empathy OR stigma OR struggle OR support OR "mental well-being" OR "mental illness" OR "emotional health" OR shame<br>)<br>AND<br>(<br>"university students" OR "higher education" OR "undergraduate students" OR "college students" OR "academic achievement" OR "student retention" OR "Health Services in Universities" OR "School leaving" OR "Student Dropouts" OR student OR university OR academic OR school OR college OR undergraduate OR persistence OR performance OR attainment OR achievement OR attrition OR dropout OR dropping out OR drop out OR fail OR failed OR failure OR GPA OR retention OR "academic health" OR success OR succeed OR skills OR rates OR competition OR dissatisfaction OR "black doctoral students" OR "graduate students" OR "college students of color" OR marginalized OR diverse OR diversity OR identities OR identity OR integration<br>)<br>AND<br>(<br>"affirmative action" OR "racial equity" OR "racism" OR "institutional racism" OR "structural racism" OR "racial discrimination" OR "ethnic discrimination" OR "minority groups" OR black OR "black students" OR African OR African-American OR color OR "students of color" OR ethnicity OR ethnicities OR ethnic OR Étnico OR Étnica OR Etnicidade OR "quota students" OR cotistas OR microaggression OR racial OR race OR "racial bias" OR "racial injustice" OR inequity OR inequality OR "health status disparities" OR resilience OR anti-blackness OR underrepresented OR diversity OR integration OR marginalized OR inclusion OR support OR barriers OR "cultural health"<br>)**

Supplementary Table 5 - Descriptors, Keywords, and "Single" Boolean Logic
